# Mediterranean diet consumption affects the endocannabinoid system in overweight and obese subjects: possible links with gut microbiome, insulin resistance and inflammation

**DOI:** 10.1007/s00394-021-02538-8

**Published:** 2021-03-24

**Authors:** Silvia Tagliamonte, Manolo Laiola, Rosalia Ferracane, Marilena Vitale, Maria A. Gallo, Victoria Meslier, Nicolas Pons, Danilo Ercolini, Paola Vitaglione

**Affiliations:** 1 Department of Agricultural Sciences, University of Naples Federico II, Parco Gussone Ed. 84, 80055 Portici, NA) Italy; 2 Department of Clinical Medicine and Surgery, University of Naples Federico II, 80131 Naples, Italy; 3 Centro Diagnostico San Ciro, 80055 Portici, Italy; 4 Université Paris-Saclay, INRAE (Institut National de Recherche Pour L’agriculture, l’alimentation Et L’environnement), MGP (Metagenopolis), 78350 Jouy en Josas, France; 5 Task Force On Microbiome Studies, University of Naples Federico II, 80134 Naples, Italy

**Keywords:** Obesity, *Akkermansia muciniphila*, Gut microbiome, Gut barrier, Cardiovascular disease risk

## Abstract

**Purpose:**

To investigate whether a Mediterranean diet (MD) affected the plasma concentrations of endocannabinoids (ECs), N-acylethanolamines (NAEs) and their specific ratios in subjects with lifestyle risk factors for metabolic diseases. To identify the relationship between circulating levels of these compounds and gut microbiome, insulin resistance and systemic inflammation.

**Methods:**

A parallel 8-week randomised controlled trial was performed involving 82 overweight and obese subjects aged (mean ± SEM) 43 ± 1.4 years with a BMI of 31.1 ± 0.5 kg/m^2^, habitual Western diet (CT) and sedentary lifestyle. Subjects were randomised to consume an MD tailored to their habitual energy and macronutrient intake (*n* = 43) or to maintain their habitual diet (*n* = 39). Endocannabinoids and endocannabinoid-like molecules, metabolic and inflammatory markers and gut microbiome were monitored over the study period.

**Results:**

The MD intervention lowered plasma arachidonoylethanolamide (AEA, *p* = 0.02), increased plasma oleoylethanolamide/palmitoylethanolamide (OEA/PEA, *p* = 0.009) and OEA/AEA (*p* = 0.006) and increased faecal *Akkermansia muciniphila* (*p* = 0.026) independent of body weight changes. OEA/PEA positively correlated with abundance of key microbial players in diet–gut–health interplay and MD adherence. Following an MD, individuals with low-plasma OEA/PEA at baseline decreased homeostatic model assessment of insulin resistance index (*p* = 0.01), while individuals with high-plasma OEA/PEA decreased serum high-sensitive C-reactive protein (*p* = 0.02).

**Conclusions:**

We demonstrated that a switch from a CT to an isocaloric MD affects the endocannabinoid system and increases *A. muciniphila* abundance in the gut independently of body weight changes. Endocannabinoid tone and microbiome functionality at baseline drives an individualised response to an MD in ameliorating insulin sensitivity and inflammation.

*Clinical Trial Registry number and website* NCT03071718; www.clinicaltrials.gov

**Supplementary Information:**

The online version contains supplementary material available at 10.1007/s00394-021-02538-8.

## Introduction

The Mediterranean diet (MD) is a healthy dietary pattern, while the Western diet is not. This idea is strongly supported by epidemiological evidence pointing out inverse associations of the two dietary patterns with the incidence of metabolic diseases, obesity and related health consequences [1, 2]. However, in the context of clinical practice the health effects that occur over a duration that is reasonable to improve intermediary clinical outcomes are not obvious. Different responses to similar diets often occur and reflect the actual status of body systems, the cross-talk ability of the involved systems and their flexibility to react to environmental (dietary) factors [3]. Randomised clinical trials (RCTs) designed to monitor multiple systems, including the gut microbiome are mandatory to determine causal relationships between diet and health outcomes with the potential to unravel physiological mechanisms underpinning the effects and obtain indications for personalised nutrition and precision medicine [4–6]. Several microbiome-targeted studies showed that MD consumption boosts fibre-degrading species and anti-inflammatory responses in the human body [7–10].

Evidence indicates that the gut microbiome can affect metabolic inflammation regulating gut epithelium permeability [11]. Specifically, the mucin-degrading bacterium *Akkermansia muciniphila* is widely considered a valuable contributor to the maintenance of gut health and metabolic homoeostasis reducing obesity and related disorders, such as glucose intolerance, insulin resistance, steatosis and gut permeability [12–14].

The endocannabinoid system has pleiotropic functions in the body, and plays a key role in the development of obesity and its comorbidities being a mediator in the relationship between the gut microbiota and host metabolism [15]. Circulating endocannabinoids (ECs) and their congeners N-acylethanolamines (NAEs) are metabolically connected to diet and are tightly involved in the regulation of energy homeostasis, appetite cues, pain sensation, inflammation, immunity, obesity and dysmetabolism [16–18]. Indeed, plasma ECs and NAEs concentrations, as well as NAEs ratios, are considered biomarkers of white adipose tissue distribution reflecting blood cholesterol and insulin resistance in obesity [18, 19] in addition to being involved in food liking and intake [20, 21]. We have recently observed several correlations between circulating NAEs, habitual diet and systemic inflammation in subjects with an ileostomy [22]. However, the effect of diet on circulating levels of ECs and NAEs in intervention studies in humans is still underexplored.

The combined high intake of slowly digestible carbohydrates, unsaturated fat, plant proteins and micronutrients in the MD as opposed to refined (quickly digestible) carbohydrates, saturated fat and animal proteins in the Western diet is responsible for the different metabolic and health effects of the two diets [23]. We recently performed an 8-week RCT in 82 overweight and obese subjects with lifestyle risk factors for metabolic disease. Forty-three subjects consumed an MD individually tailored to each participant’s habitual energy and macronutrient intake (MD group), and 39 subjects in the control group (CT) maintained their habitual diet that was very similar to a Western diet. A *per-protocol analysis* of data, excluding 18 participants, showed that the MD group diminished plasma cholesterol and exhibited some changes in the gut microbiome and related metabolome [9].

In this study, we explore the impact of an isocaloric dietary shift from a Western diet to an MD on the circulating levels of ECs, NAEs, oleoylethanolamide/arachidonoylethanolamide (OEA/AEA) and oleoylethanolamide/palmitoylethanolamide (OEA/PEA) ratios. Further, by performing an *intention-to-treat analysis* of data, we shed light on the relationships of the endocannabinoid system with the gut microbiome, clinical data and adherence to MD.

## Methods

### Study design and participants

This study is part of a randomised and controlled eight-week intervention trial whose primary outcome was to detect changes in fasting plasma lipids and faecal levels of short-chain fatty acids upon an intervention with MD, as described in a previous paper [9]. That paper also reported the details of the study design and protocol [9]. In this paper for the first time, we focused on secondary outcome, such as plasma ECs and NAEs, OEA/AEA and OEA/PEA ratios. Specifically, we analysed their changes in response to the dietary intervention, concomitant with gut microbiome changes and their correlations with variables related to diet (food categories, nutritional composition and adherence to the Mediterranean diet), insulin resistance (Homeostatic Model Assessment for Insulin Resistance, HOMA-IR [24] and systemic inflammation (serum high-sensitive C-reactive protein, hs-CRP). The study was conducted after the approval of the University of Naples Ethic Committee (Protocol number: 108/16). The trial was registered at www.clinicaltrials.gov (number NCT03071718). Recruitment was performed at the Department of Agricultural Sciences and at the Department of Clinical Medicine and Surgery of the University of Naples, Naples, Italy.

The 334 potentially eligible adults, interested in study participation, were screened on the basis of the inclusion/exclusion criteria considering medical and lifestyle conditions [9]. Briefly, subjects were included if they were healthy men or women aged between 20 ≤ age ≤ 65 years, with BMI ≥ 24 kg/m^2^, consuming a diet characterised by an intake of fruits and vegetables ≤ 3 servings/day, no consumption of probiotics, functional foods and/or food supplements, consuming no more than 2 portions a day of whole-grain food and/or enriched with dietary fibre, having a sedentary lifestyle (MET min/week < 700). Informed written consent was obtained prior to undertaking the study. Eighty-two participants were randomised between the two intervention arms of the parallel designed study and completed the study protocol. The characteristics of the participants and variations over the intervention of many clinical outcomes are reported in the Online Supplementary Table 1 by Meslier and co-workers [9].

**Table 1:** Daily food intake (g) within each category in the control (CT) and Mediterranean diet group (MD) at baseline (0 wk), 4 weeks (4 wk) and 8 weeks (8 wk)

g/day	CT (*n* = 39)			MD (*n* = 43)			*p* values		
	0 wk	4 wk	8 wk	0 wk	4 wk	8 wk	Δ4-0	Δ8-0	Δ8-4
Fruit ^d^	139.5 ± 22.0	195.0 ± 33.5	183.9 ± 30.9	171.3 ± 21.8^b^	275.6 ± 25.5^a^	271.7 ± 22.2^a^	0.141	0.014^*^	0.919
Vegetables	179.3 ± 16.7	190.4 ± 13.5	174.7 ± 16.1	203.9 ± 15.7^b^	298.0 ± 22.5^a^	284.4 ± 23.1^a^	0.010^*^	0.006^*^	0.930
Legumes	14.1 ± 2.4	20.7 ± 3.4	15.9 ± 3.2	14.2 ± 2.5^b^	48.3 ± 6.5^a^	50.0 ± 8.5^a^	< 0.001^*^	< 0.001^*^	0.240
Whole-grain products	5.6 ± 2.2	4.4 ± 2.0	5.7 ± 3.0	13.9 ± 6.1^b^	148.8 ± 21.5^a^	147.6 ± 21.9^a^	< 0.001^*^	< 0.001^*^	0.902
Refined-grain products	143.6 ± 11.1	141.9 ± 19.0	142.9 ± 18.2	70.7 ± 9.2^a^	37.2 ± 7.3^b^	26.4 ± 5.0^b^	< 0.001^*^	0.019^*^	0.241
Eggs	10.8 ± 2.1^**⁑**^	13.3 ± 5.0	8.2 ± 1.8	4.5 ± 1.1^a**⁑**^	1.7 ± 0.8^b^	2.0 ± 0.7^b^	0.136	0.823	0.040^*^
Dairy products	94.3 ± 14.9^b^	108.9 ± 5.9^a^	93.3 ± 14.0^ab^	83.2 ± 13.9	91.5 ± 14.2	96.2 ± 15.4	0.192	0.673	0.452
Fish products	29.1 ± 5.0	30.2 ± 4.8	33.3 ± 5.0	26.1 ± 5.6^c^	49.2 ± 6.2^a^	40.3 ± 6.0^b^	0.002^*^	0.071	0.006^*^
Meat	75.3 ± 9.2^b^	63.2 ± 7.2^a^	72.2 ± 8.4^ab^	55.5 ± 8.2^a^	11.6 ± 2.3^b^	16.9 ± 4.6^b^	0.003^*^	0.001^*^	0.896
Oils and fats	6.6 ± 2.1	7.5 ± 2.1	6.7 ± 2.0	2.5 ± 0.7^a^	0.3 ± 0.2^b^	1.8 ± 0.9^a^	< 0.001^*^	0.021^*^	0.264
Coffee	53.7 ± 8.4	51.9 ± 7.8	52.5 ± 10.2	44.5 ± 8.0^b^	58.7 ± 8.3^a^	50.7 ± 7.2^a^	0.059	0.215	0.700
Fruit juices	11.8 ± 3.6^a^	7.7 ± 5.1^b^	11.7 ± 5.9^ab^	3.6 ± 1.5	3.7 ± 2.6	4.6 ± 2.7	0.048^*^	0.046^*^	0.618
Wine	8.8 ± 1.8	9.6 ± 3.1	4.1 ± 1.5	24.5 ± 5.1	18.8 ± 8.2	19.9 ± 8.3	0.632	0.128	0.477
Snacks	76.2 ± 13.9	76.6 ± 11.3	73.7 ± 13.0	44.8 ± 6.9^a^	20.2 ± 5.2^b^	24.2 ± 5.1^b^	0.011^*^	0.009^*^	0.492
Soft drinks	46.9 ± 12.4	33.7 ± 12.0	35.1 ± 10.8	31.7 ± 15.9	35.6 ± 23.3	18.4 ± 9.7	0.730	0.507	0.810
IMI^e^	4.5 ± 0.3^a^	4.1 ± 0.3^a^	3.8 ± 0.2^b^	5.2 ± 0.3^b^	8.0 ± 0.3^a^	7.8 ± 0.3^a^	< 0.001^*^	< 0.001^*^	0.664

Data are expressed as the mean ± SEM. Different letters on a row indicate significant differences between time points within groups by Wilcoxon test or 2-way ANOVA repeated measures depending on normal distribution of the data. * p < 0.05 pairwise time points (Δ) between CT and MD by Mann–Whitney test or independent-sample T test

^⁑^*p* < 0.05 between MD and CT at baseline by Mann–Whitney test adjusted for energy intake

^d^Fruits: sum of fruit and nuts;

^e^IMI, Italian Mediterranean Index

### Dietary intervention

MD participants received an isocaloric tailored MD diet based on their habitual energy and macronutrient intake assessed by a 7-day Food Diary, while CT volunteers were asked not to change their habitual diet during the 8-week intervention. Every 2 weeks, the individual compliance was assessed through food diaries, whereas adherence to the Mediterranean diet was assessed by Italian Mediterranean Index (IMI) [9].

### Anthropometric measurement and biochemical analysis

A trained operator measured the waist circumference midway between the lowest rib and the iliac crest using an anthropometric tape. Hip circumference was measured around the widest portion of the buttocks, with the tape parallel to the floor. Weight from subjects wearing light clothes was measured, after voiding, to the nearest 0.1 kg on a digital scale (Model 703; Seca). Height of subjects was recorded to the nearest 0.5 cm with a stadiometer (Model 213; Seca). Body composition, including total body water, fat mass and fat-free mass, was determined at an ambient temperature after voiding and after being in a supine position for at least 20 min, through an electric impedance analysis with a single-frequency 50-kHz bioelectrical impedance analyser (BIA 101 RJL; Akern Bioresearch) as described in Meslier and co-workers [9]. Body mass index (BMI) was calculated as weight in kg divided by height in meters squared. According to the World Health Organisation (WHO) task force and the National Institutes of Health (NIH) guidelines, people whose BMI is between 18.5 and 24.9 kg/m^2^ are considered normal weight, between 25 and 29.9 kg/m^2^ are overweight and > 30 kg/m^2^ are obese.

Fasting blood samples were collected by venipuncture into a serum separator, EDTA-containing tubes and EDTA aprotinin-containing tubes. All EDTA blood samples were kept chilled/on ice before processing. Plasma samples were prepared by centrifugation at 3000 rpm for 15 min at 4 °C and within 15 min of collection. All biological samples were rapidly frozen and stored at − 80 °C until analysis. Plasma glucose, insulin and serum hs-CRP concentrations as well as HOMA index were assessed as described previously [9].

### Extraction of ECs and NAEs from plasma samples

The analysis of ECs and NAEs was performed in EDTA plasma samples. All samples were thawed in the fridge at 4 °C before extraction and samples were kept chilled on ice during the specific extraction procedures. A solid-phase extraction (SPE) according to the method described by Marczylo and colleagues was carried out [25].

Plasma samples (500 µL) previously diluted 1:2 with distilled water were added with 50 µL of the internal standard 200 ng/mL solution of Arachidonoylethanolamide d8 (AEA d8) (Cayman Chemical, Ann Arbor, MI). Then, the samples were vortexed and centrifuged 21,100*g* for 5 min at 4 °C.

Oasis HLB cartridges (1 cc/30 mg Waters) were preconditioned with 1 mL of methanol and equilibrated using 1 mL of H_2_O. Samples were introduced onto the cartridges and were washed with 1 mL of aqueous methanol (40%), and the monitored compounds were eluted in 1 mL of acetonitrile. The eluate was dried under nitrogen flow and reconstituted in 100 µL acetonitrile/water (50:50) before the LC–MS/MS analysis.

### LC–MS/MS analysis

Chromatographic separation was performed using an HPLC apparatus provided with two micropumps, Perkin-Elmer Series 200 (Norwalk, CT, USA). The compounds were separated on a Synergi Max RP 80 column (50 × 2.1 mm) (Phenomenex, USA) with a setting temperature of 30 °C and a flow rate of 0.2 mL/min. The injection volume was 10 μL. The monitored compounds were eluted by a linear gradient of H_2_O and 0.2% formic acid (solvent A) and CH_3_CN (solvent B). According to Mennella and co-workers [21], the eluting gradient was set as follows: 50–79% B (10 min), 79–95% B (1 min), constant at 95% B (2 min) and finally returning to the initial conditions within 2 min. The acquisition was performed in positive ion mode on an API 3000 triple quadrupole mass spectrometer (Applied Biosystems, Canada) in MRM (Multiple Reaction Monitoring). All the acquisition parameters are summarised in Supplementary Table 1.

ECs (2-arachidonoylglycerol, 2-AG; anandamide, AEA; AEAd8) and NAEs (oleoylethanolamide, OEA; linoleoylethanolamide, LEA; palmitoylethanolamide, PEA) standards were purchased from Cayman (Cayman Chemical, Ann Arbor, MI).

The limit of detection (LOD) and limit of quantification (LOQ) of the identified molecules are reported in Supplementary Table 2.

### Faecal microbiome determination by shotgun metagenomics

A full description of the sampling, sequencing and data analysis procedures is reported in the online supplementary material by Meslier and colleagues [9]. Briefly, faecal DNA extraction was performed following IHMS SOP P7 V2 and purified DNA fragment libraries were sequenced using the Ion Proton Sequencer (ThermoFisher Scientific, Waltham, US) with a minimum of 20 million 150-bp high-quality reads generated per library. Metagenomic Species Pangenome (MSP) abundance profiles were determined and annotated as previously described [9, 26] and the in-house FAnToMet pipeline was used to assess the functional potential modules of the intestinal gut microbiome.

### Quantification of Akkermansia muciniphila

The abundances of *A. muciniphila* in each sample were further quantified and analysed through qPCR as described by Dao et al. [27]. Briefly, extracted DNA was quantified using Qubit Fluorometric Quantitation (ThermoFisher Scientific, Waltham, US) and amplified using primers targeting the 16S rRNA for *A. muciniphila* (forward CAGCACGTGAAGGTGGGGAC and reverse CCTTGCGGTTGGCTTCAGAT). The total 16S rRNA gene was also quantified and used as reference gene in order to normalise the abundance of *A. muciniphila* in all samples tested (bacterial universal primers forward ACTCCTACGGGAGGCAGCAG and reverse ATTACCGCGGCTGCTGG). Amplifications were performed using the StepOne Real-Time PCR System (Life Technologies) with the following thermal cycling profiles: one cycle at 95 °C for 30 s, 40 cycles at 60 °C for 30 s and 72 °C for 30 s and using the SYBR Green PCR Master Mix Reagents Kit (Thermo Fisher Scientific). Negative controls (H_2_O) were included in each qPCR run and each assay was performed in triplicate. The results of the amplifications were expressed as threshold cycle (Ct) values. The relative quantification of *A. muciniphila* in all samples was calculated using the comparative Ct method (ΔCt, the difference between the Ct value of *A. muciniphila* and the Ct of total 16S rRNA gene for each sample). Lastly, the concentration of *A. muciniphila* was carried out by comparing ΔCt measures with a standard calibration curve (Y = − 3.527 × log10(X) + 17.677, R2 = 0.97, PCR efficiency = 92.11%) and by relating such values to total faecal DNA concentration.

### Statistical analysis

A power analysis using the mean plasma concentrations and variations of ECs and NAEs in previous studies was performed to estimate the sample size needed to detect an effect of diet on plasma concentrations of ECs and NAEs [19, 28]. We calculated that 39 participants per group was sufficient to detect a minimum inter-group difference of 23% and 13% in plasma concentrations of 2-AG and AEA, 10% and 14% in plasma concentrations of PEA and OEA as well as 10% and 9% in AEA/OEA and OEA/PEA ratios, respectively, with an α error of 0.05, 80% power and 2-sided testing. Therefore, 39 participants in CT and 43 in MD would be adequate to test the effect of MD on fasting circulating levels of ECs and NAEs.

All statistical analyses were performed in R version 3.6.0. After being checked for normality, variables showing a significant positive skewness, were transformed in ln (x). For those variables that showed a normal distribution at the Kolmogorov–Smirnov test, Independent samples T test was performed to check differences between CT and MD at baseline. The 2-way ANOVA with repeated measure was performed to check within group differences over the study period. For those variables, which did not show a normal distribution after logarithmic transformation, a non-parametric Wilcoxon test was performed. To test the overall correlation among ECs, NAEs, microbiome and dietary variables, pairwise Spearman's rank correlations within CT and MD at baseline, 4 and 8 weeks were calculated and adjustments were performed using the Benjamini–Hochberg procedure and Spearman rho values were filtered by keeping correlations with at least one false discovery rate (FDR) of ≤ 0.05. The heatmap of correlation was visualised using the Hmisc package and the function heatmap.2. Two-tailed P values lower than 0.05 were considered significantly different. The data are expressed as means ± SEM.

Differently abundant species between MD and CT groups at either 4 or 8 weeks of dietary intervention were assessed through test relations function of momr R package [29], as well as gut microbial modules differences at baseline between quartiles.

The linear discriminant analysis (LDA) effect size (LEfSe) [30] was used to identify differentially abundant species between Q1 and Q4 groups, for a *p* < 0.05 after the Kruskal–Wallis test. Only discriminative features with an LDA score > 2.0 were plotted and ranked by effect size.

## Results

### Mediterranean diet decreases plasma anandamide concentrations

The 82 participants (43 F and 39 M, average body mass index (BMI) 31.1 ± 0.5 kg/m^2^, age 43 ± 1.4 years), of which 43 participants (22 F/21 M, average BMI 30.9 ± 0.6 kg/m^2^, age 43 ± 1.9 years) were in MD group and the 39 participants (21 F/18 M, average BMI 31.2 ± 2.0 kg/m^2^, age 42 ± 2.0 years) in CT group, showed high compliance with the intervention.

The intake of the main food categories monitored over the study period is reported in Table 1. The intakes were homogeneous between the two groups at baseline for all food categories except eggs which were consumed more by the participants in CT. Participants in MD decreased refined-grain products, snacks, oils, fats and meat intake over the study period and increased their intake of vegetables, legumes, whole-grain products and fish products after 4 and 8 weeks, whereas fruit and nuts after 8-week intervention. These changes were coherent with an increased adherence to the MD as assessed by the Italian Mediterranean Index (IMI).

No significant effect of gender on baseline plasma concentration of OEA, LEA, PEA, AEA, 2-AG and OEA/PEA and OEA/AEA ratios in CT and MD group was found.

**Fig. 1:**
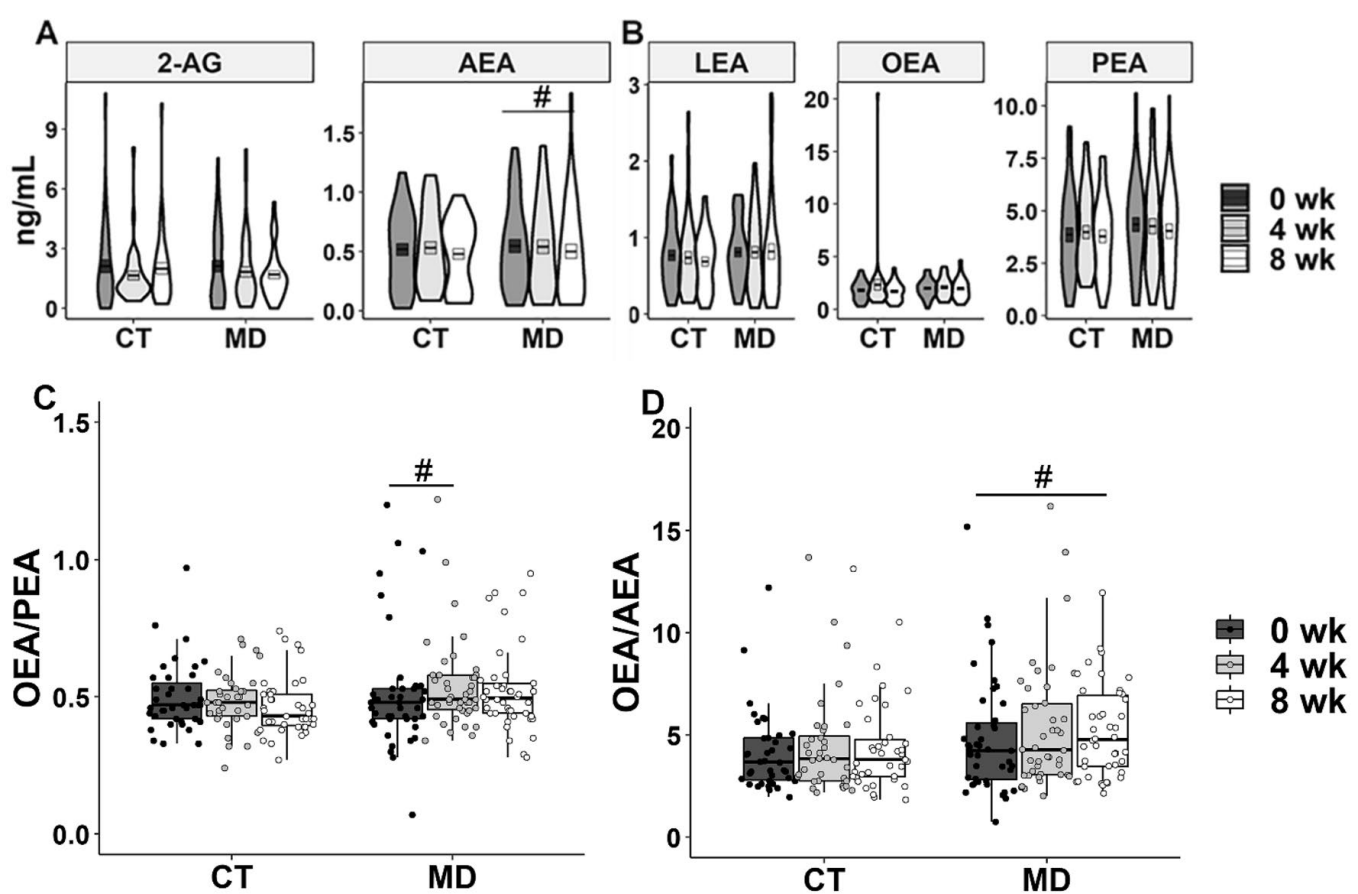
Violin plots showing the distribution of plasma endocannabinoids (**a**), concentrations of N-acylethanolamines (**b**), and box plots showing the plasma OEA/PEA ratio (**c**) and the OEA/AEA ratio (**d**) in participants from the control group (CT) and the Mediterranean diet group (MD) at baseline (0 wk), 4 weeks (4 wk) and 8 weeks (8 wk). # *p* < 0.05 within group by Wilcoxon test. In C and D, two outliers were removed for simpler visualisation. 2-AG, 2-Arachidonoylglicerol; AEA, Arachidonoylethanolamide; LEA, Linoylethanolamide; OEA, Oleoylethanolamide; PEA, Palmitoylethanolamide

MD consumption lowered plasma AEA concentrations after 8 weeks compared to the baseline concentrations (-9.1%; *p* = 0.02), while it did not affect 2-AG, linoleoylethanolamide (LEA), OEA or PEA (Fig. 1a, b). Interestingly, significant increases in the OEA/PEA ratio (5.9%; *p* = 0.009) and the OEA/AEA ratio (14.6%; *p* = 0.006) were registered after 4 and 8 weeks of MD consumption, respectively (Fig. 1c, d). Moreover, negative associations of plasma OEA/AEA with waist circumference (Spearman’s rho = − 0.462, *p*  ≤ 0.001) and hip circumference (Spearman’s rho = − 0.200, *p* = 0.002) were observed.

### Higher Akkermansia muciniphila correlates with plasma AEA concentration after the MD intervention

**Fig. 2:**
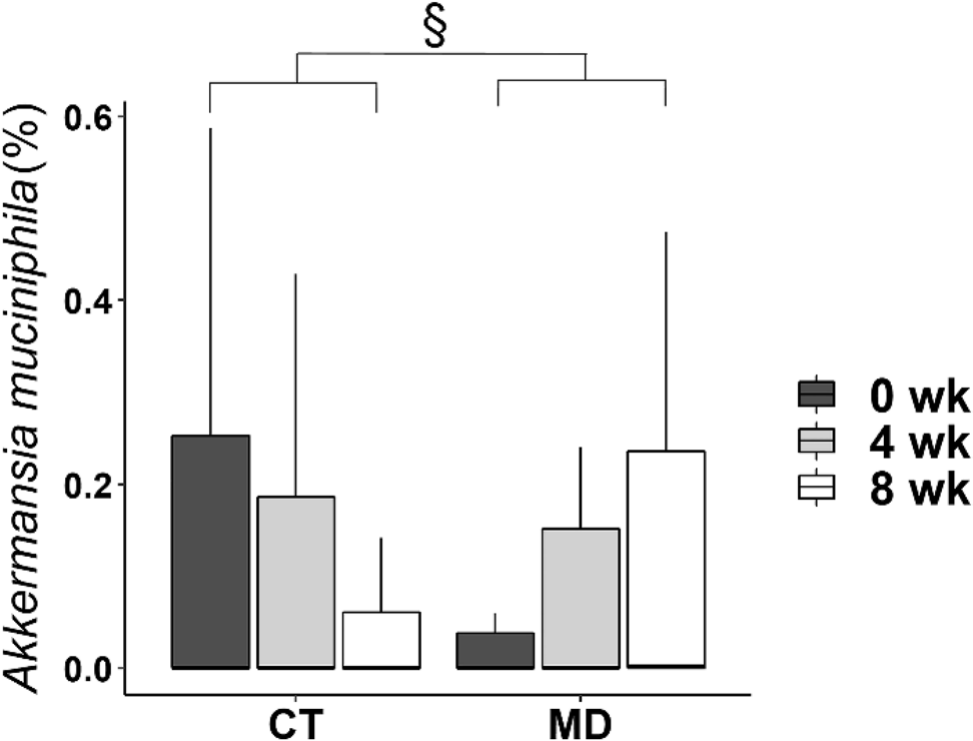
Faecal *Akkermansia muciniphila* relative abundance calculated from shotgun metagenomics in participants from the control group (CT) and Mediterranean diet group (MD) at baseline (0 wk), 4 weeks (4 wk) and 8 weeks (8 wk). § *p* < 0.05 pairwise time points (Δ) between CT and MD by the Mann–Whitney test

**Fig. 3:**
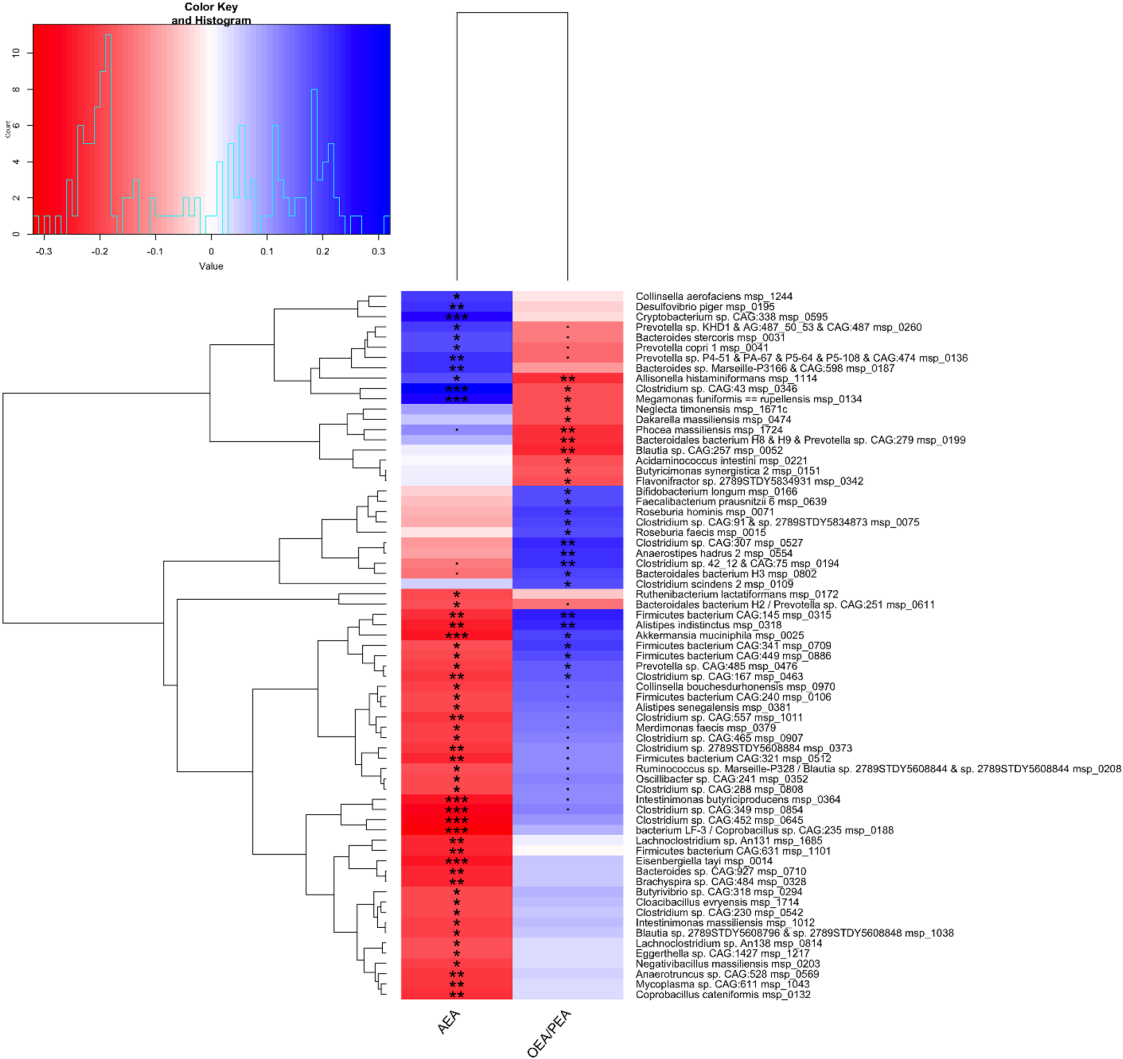
Heatmap showing hierarchical ward-linkage clustering of AEA and the OEA/PEA ratio based on Spearman’s correlation with gut microbiome species. The colour scale represents the magnitude of Spearman’s rho coefficient, with red indicating negative correlations and blue indicating positive correlations. Adjustments were performed using the Benjamini–Hochberg procedure, and Spearman’s rho values were filtered by maintaining correlations with at least one false discovery rate (FDR) of < 0.05. ▪, FDR = 0.05; *, FDR < 0.05; **, FDR < 0.01; ***, FDR < 0.001. AEA, Arachidonoylethanolamide; OEA, Oleoylethanolamide; PEA, Palmitoylethanolamide

Consistently with the results reported in our previous paper and obtained by analysing a *per protocol* selection of the population, the isocaloric MD dietary intervention led to gut microbiome changes in the overall population [9]. Indeed, we observed some MSP species, such as *Roseburia faecis* and *R*. *hominis* significantly enriched in MD as compared to CT group, after either 4 or 8 weeks, along with an increase in the fibre-degrading *Faecalibacterium prausnitzii* and several members of the *Lachnospiraceae* taxa, while *Ruminococcus torques* and *R*. *gnavus* were CT-related species (Supplementary Tables 3 and 4). Interestingly, significantly higher levels of *Akkermansia muciniphila* occurred in the MD as compared to CT group after 8 weeks of MD consumption (*p* < 0.05, Fig. 2), although this was not observed in the previous *per protocol* analysis [9]. Later, we further investigated and confirmed the identification of *A. muciniphila* through a quantitative real-time polymerase chain reaction (qPCR) (Spearman’s Rho = 0.8, p value = 2.2e-16, Supplementary Fig. 1). Plasma concentrations of OEA (Spearman’s rho = -0.295, p =  < 0.001), PEA (Spearman’s rho = -0.341, p =  < 0.001), LEA (Spearman’s rho = -0.366, p =  < 0.001) and AEA (Spearman’s rho = -0.319, *p* value =  < 0.001) were negatively correlated with *A. muciniphila*, which was positively linked to the plasma OEA/PEA (Spearman’s rho = 0.180, *p* = 0.005) and OEA/AEA ratios (Spearman’s rho = 0.259, *p* < 0.001) (Fig. 3). Plasma AEA concentrations were negatively associated with *Eggerthella* sp. Furthermore, circulating levels of AEA and the OEA/PEA ratio showed opposite associations with the abundance of *Intestinimonas butyriciproducens*, *Bifidobacterium longum*, *Roseburia hominis* and *Faecalibacterium prausnitzii, Prevotella* sp*.* and *P. copri.* All the correlations found are detailed in Fig. 3.

### Plasma OEA/PEA ratio is associated to MD adherence

**Fig. 4:**
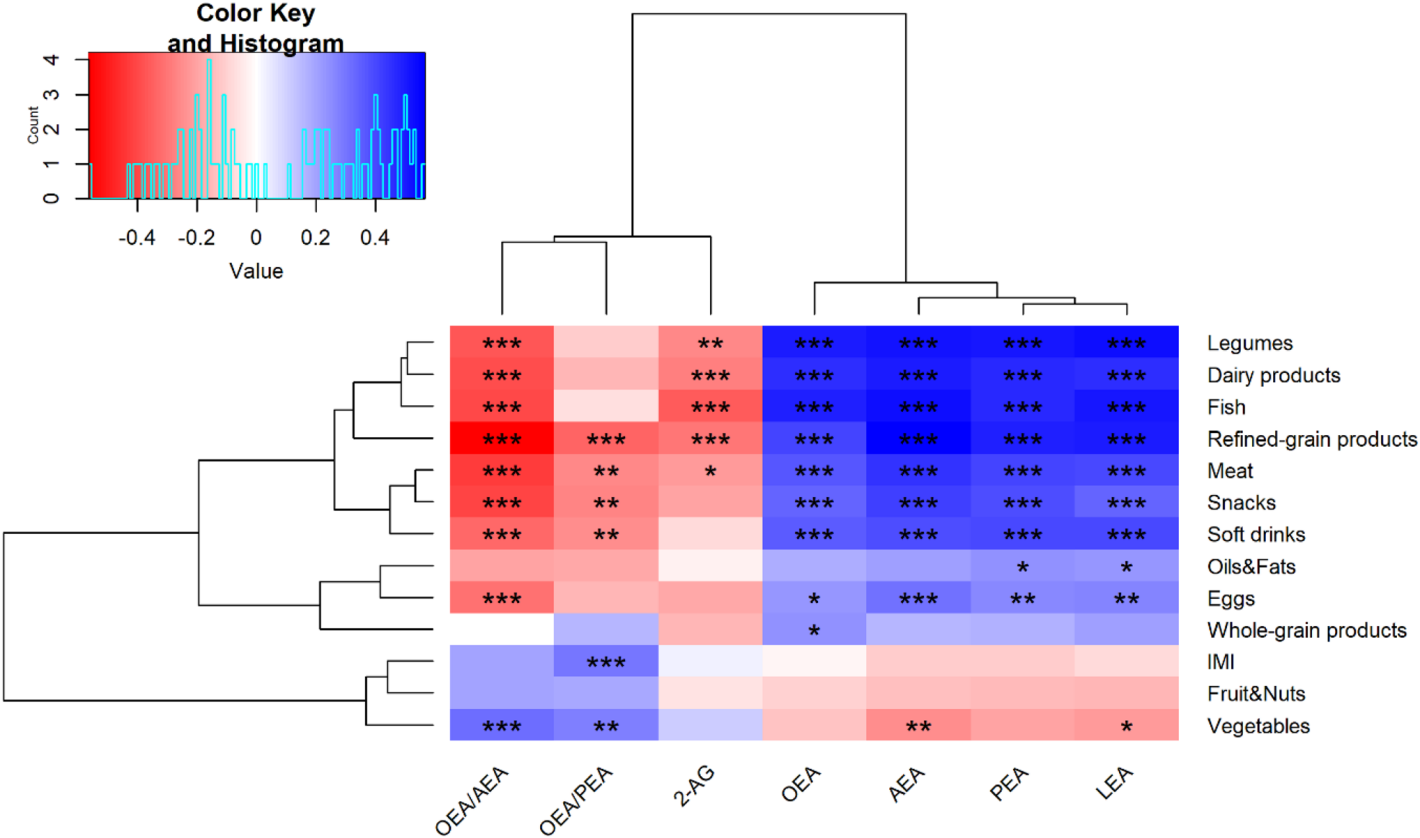
Heatmap correlation matrix between plasma ECs, NAEs, OEA/PEA ratio, OEA/AEA ratio and food categories and IMI. The colour scale represents the magnitude of Spearman’s rho coefficient, with blue indicating a positive correlation and red indicating a negative correlation. * p < 0.05; ** p < 0.01; *** *p* < 0.001 significant Spearman’s correlations and Holm correction adjusted for Energy intake. IMI, Italian Mediterranean Index; 2-AG, 2-Arachidonoylglicerol; AEA, Arachidonoylethanolamide; LEA, Linoylethanolamide; OEA, Oleoylethanolamide; PEA, Palmitoylethanolamide

OEA/PEA ratio, but not OEA/AEA ratio, was positively correlated with IMI. OEA/PEA ratio also showed positive correlation with daily intake of vegetables and negative correlation with soft drinks, snacks, meat and refined-grain products intake (Fig. 4). In addition, OEA/AEA was correlated negatively with eggs, soft drinks, snacks, meat and refined-grain products, fish, dairy products and legumes intake. While plasma 2-AG concentrations showed negative correlations with meat, refined-grain products, fish, dairy products and legumes intake, which conversely, were positively correlated with plasma AEA and NAEs concentrations.

### Baseline plasma OEA/PEA ratio influences the individual response to the consumption of an MD

**Fig. 5:**
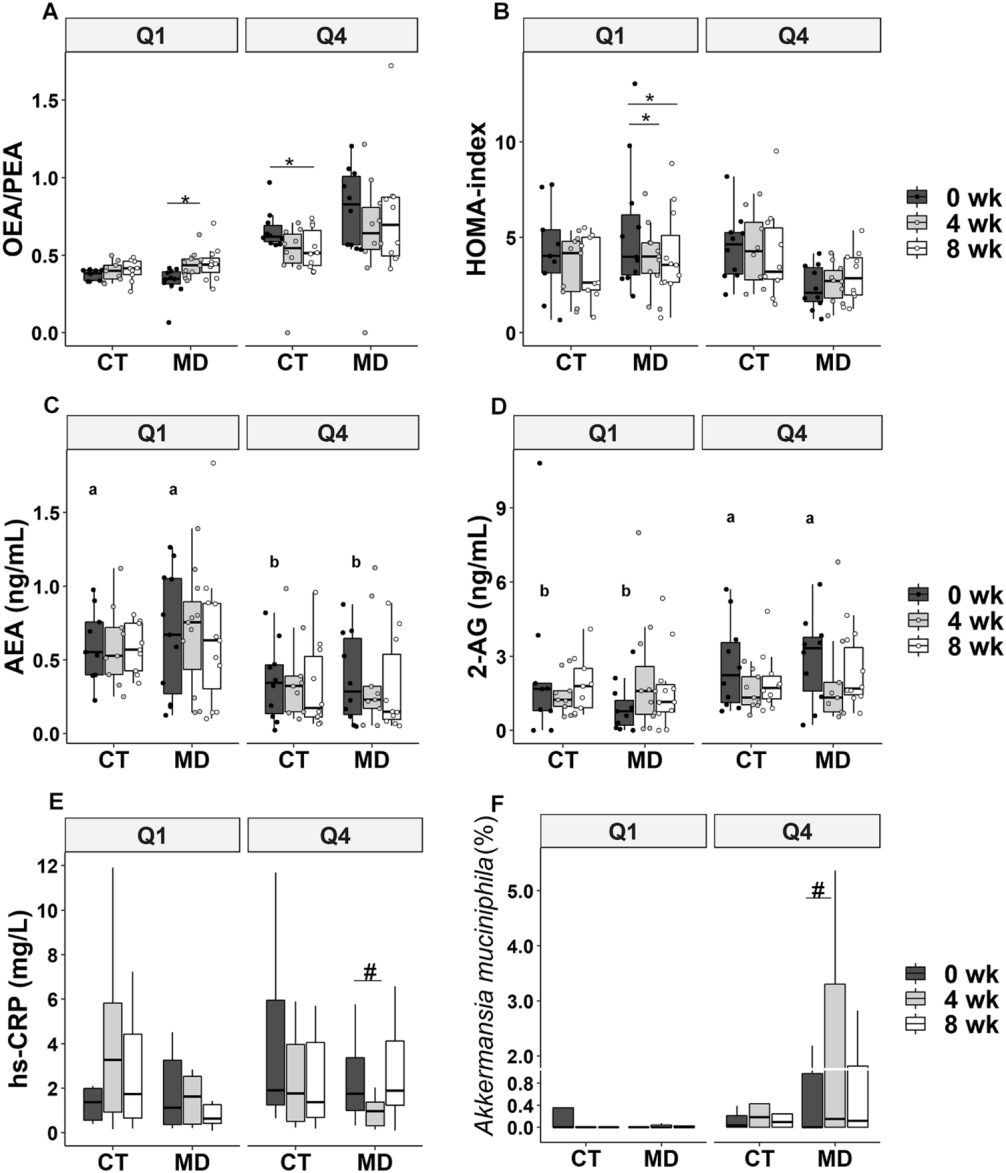
Plasma OEA/PEA ratio (**a**), HOMA index (**b**), plasma AEA concentration (**c**), plasma 2-AG concentration (**d**), serum hs-CRP concentration (**e**) and faecal *Akkermansia muciniphila* relative abundance (**f**) in participants from the control group (CT) and Mediterranean diet group (MD) in the lowest (Q1) and highest quartile (Q4) for OEA/PEA ratio at baseline (0 wk), 4 weeks (4 wk) and 8 weeks (8 wk). Q1 = minimum to 25th percentile of OEA/PEA ratio at baseline (CT, *n* = 9; MD, *n* = 11), Q4 = 75th percentile to maximum of OEA/PEA ratio at baseline (CT, *n* = 10; MD, *n* = 10). Different letters on the box plots indicate *p* < 0.05 between quartiles (MD + CT) at 0 wk by independent samples T test. *p < 0.05 within-group comparison by 2-way ANOVA with repeated measures; ^#^*p* < 0.05 within-group comparison by Wilcoxon test. Two outliers have been removed from panel E and 1 outlier from panel F for simpler visualisation. 2-AG, 2-Arachidonoylglicerol; AEA, Arachidonoylethanolamide; OEA, Oleoylethanolamide; PEA, Palmitoylethanolamide; hs-CRP, high-sensitivity C-reactive protein; HOMA index, Homeostatic model assessment of insulin resistance index

To test the hypothesis of whether the baseline OEA/PEA ratio affected the individual response to MD consumption, we grouped the population into quartiles of the baseline OEA/PEA ratio. MD participants in the lowest quartile (Q1) exhibited a significantly increased OEA/PEA ratio after 4 weeks (26.7%, *p* = 0.04), while those in the highest quartile (Q4) did not exhibit a change in the OEA/PEA ratio over the study period (Fig. 5a). Interestingly, Q1 participants in the MD group also exhibited a diminished HOMA index after 4 (− 26.2%, *p* = 0.01) and 8 weeks (-23.9%, *p* = 0.01) (Fig. 5b).

When compared with participants in Q1, those in Q4 displayed lower plasma PEA, LEA and AEA concentrations at baseline, which remained stable during the study period, and higher plasma 2-AG concentrations; plasma OEA did not differ at baseline between participants in Q1 and Q4 and did not change over the course of the intervention (Supplementary Fig. 2 and Fig. 5c, d).

MD participants in Q1 had increased circulating 2-AG levels after 4 weeks (121%; *p* = 0.02) (Fig. 5d), while those in Q4 had decreased serum hs-CRP (− 62.6%; *p* = 0.02) (Fig. 5e) and increased faecal abundance of *A. muciniphila* (Fig. 5f).

**Fig. 6:**
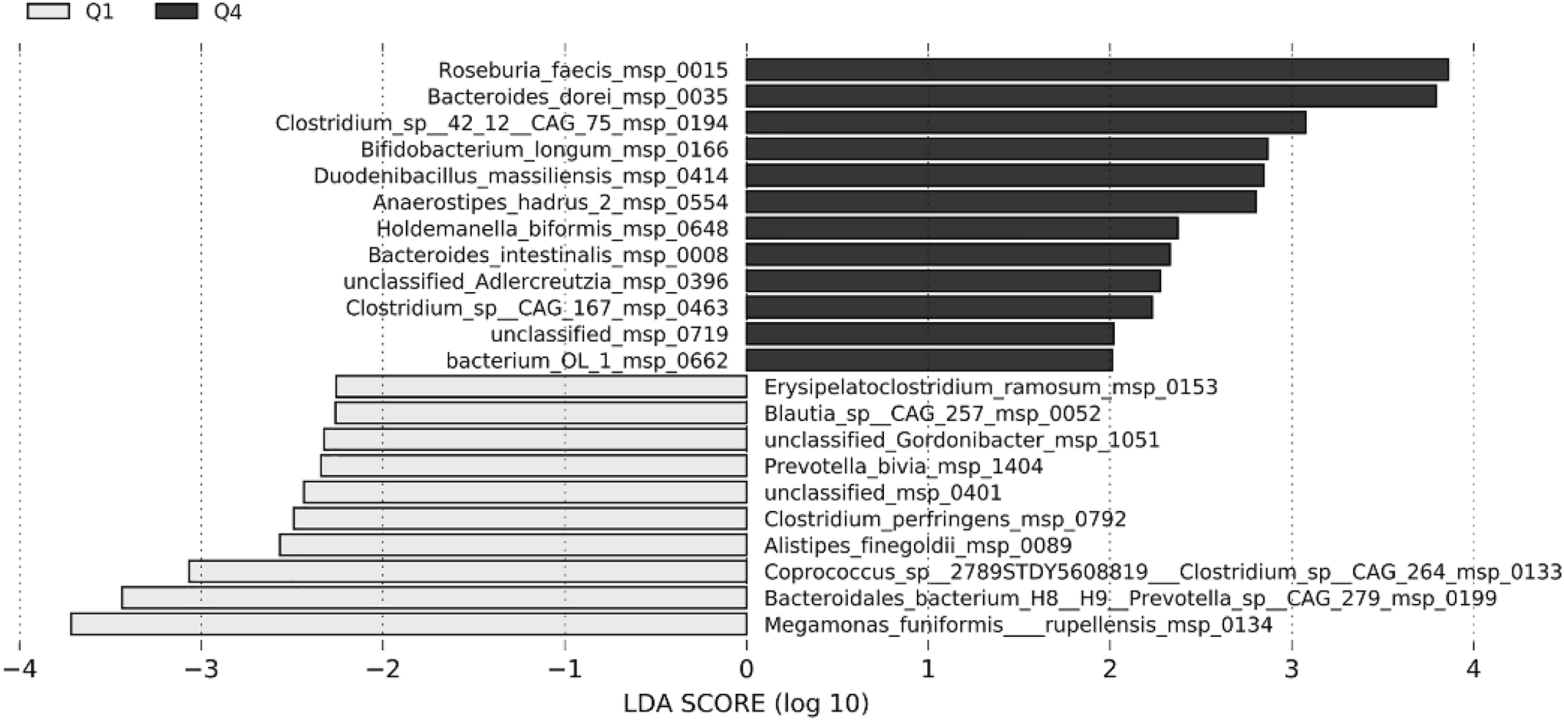
Linear discriminant analysis effect size (LEfSe) showing species that were differentially abundant between Q1 (grey) and Q4 (black). Logarithmic linear discriminant analysis (LDA) score > 2, *p* < 0.05. Q1 = minimum to 25th percentile of OEA/PEA ratio at baseline (CT, *n* = 9; MD, *n* = 11), Q4 = 75th percentile to maximum of OEA/PEA ratio at baseline (CT, *n* = 10; MD, *n* = 10)

In Fig. 6 are detailed the microbiome species that differed between Q1 and Q4 at baseline. In particular, as compared to Q1 participants, Q4 participants at baseline harboured higher levels of *Roseburia faecis, Bacteroides dorei, Bacteroides intestinalis, Bifidobacterium longum* and *Anaerostipes hadrus*, *Clostridium* sp. and lower *Blautia, Coprococcus*, *Alistipes* and *C. ramosum* in the gut (Fig. 6).

We further explored the functional and metabolic features of microbial communities in Q1 and Q4 participants at baseline (Supplementary Table 5). Q4 participants showed more abundant levels of glycocholate (MF0044), glucose (MF0057) and lactose (MF0048) degradation modules.

### Diet composition influences plasma OEA/PEA ratio at baseline

Q1 and Q4 participants at baseline showed no different intake of energy, dietary fibres, fats, saturated fatty acids, monounsaturated fatty acids, polyunsaturated fatty acids, carbohydrates, proteins, sugars and alcohol as well as anthropometric variables and body composition (Supplementary Table 6). In contrast, Q1 participants at baseline consumed more refined-grain products, meat, oils and fats, snacks than Q4 participants (Supplementary Table 7). During the MD intervention, Q1 participants significantly increased their fruit, vegetables, whole-grain products, legumes, fish intake and decreased the intake of refined-grain products, oils and fats, meat and snacks. Q4 participants in the MD group consumed more whole-grain products and less refined-grain products than those in the CT group and decreased snacks and eggs intake only after 4 weeks.

## Discussion

In this study, we showed that an isocaloric dietary shift from a Western diet to an MD lowered plasma AEA concentration and increased the OEA/PEA and OEA/AEA ratios. The increased total polyunsaturated fatty acid intake and decreased saturated fatty acid intake caused by increased consumption of fish products and nuts as isocaloric replacements of meat and dairy products might explain the changes in the circulating lipid mediators observed in MD participants [9, 31, 32]. No previous evidence of a food- or diet-induced change in the plasma OEA/PEA or OEA/AEA ratio is available. Interestingly, the OEA/PEA and OEA/AEA ratios are markers of blood cholesterol and insulin resistance [19], and their increase occurred concomitantly with the reduction in plasma cholesterol in our cohort [9].

On the other hand, mounting evidence in obese subjects and patients with inflammatory bowel disease (IBD) indicates that circulating AEA is a mediator of the effects of obesity on gut barrier integrity, intestinal permeability and inflammation [33, 34] that underpin metabolic endotoxaemia [35]. Therefore, the reduction of AEA in the MD group suggests that MD may exert metabolic and anti-inflammatory effects through its action on intestinal permeability and endocannabinoid signalling.

This hypothesis was supported by the significant increase of *A. muciniphila* faecal abundance after 8 weeks of MD compared to CT. *A. muciniphila* was not among the microbial species found to change upon intervention in the subgroup of subjects previously analysed that excluded 20 participants because of a slight increase of fruit and vegetable consumption over the run-in period when compared with the consumption at the time of enrolment [9].

*A. muciniphila* is widely considered a valuable contributor to the maintenance of gut health and metabolic homoeostasis [36]. Indeed, *A. muciniphila* abundance decreases in several pathological conditions where intestinal dysbiosis, gut barrier functionality and endotoxaemia play key roles, such as obesity, type 2 diabetes, IBD and hypertension [27, 37, 38]. Contrarily, *A. muciniphila* abundance increases following pharmacological treatments, bariatric surgery and prebiotic-based interventions that ameliorate multiple aspects of nutrient metabolism and gut barrier function [12, 27, 39, 40].

Therefore, we consider the increased abundance of *A. muciniphila* independently of body weight change as an indicator of the alleviation of obesity-related gut barrier dysfunction.

In addition, circulating levels of AEA were negatively and OEA/PEA ratio was positively correlated with a number of other microorganisms associated with a healthy gut, such as *Intestinimonas butyriciproducens*, *Bifidobacterium longum*, *Roseburia hominis* and *Faecalibacterium prausnitzii* [41–43], or known to metabolise ellagic acid and produce anti-inflammatory urolithins, such as *Eggerthella* [9]. Similar findings were recorded for the abundance of *Prevotella* sp*.* and *P. copri*, which show contradictory association with either health conditions or intestinal and metabolic diseases [44, 45], likely due to different genetic traits of the microrganisms according to strain diversity and dietary patterns [44, 46].

Altogether, these findings suggest that plasma AEA concentration may be a putative marker of altered gut permeability and dysbiosis, as already shown for intestinal AEA concentrations [15]. Contrarily, the OEA/PEA ratio may be an indicator of healthy gut barrier function. Indeed, participants falling in the quartile for the highest plasma levels of OEA/PEA (Q4) harboured in the gut higher abundances of fibre- or protein-degrading bacteria and species positively associated with a healthy gut barrier due to the production of short chain fatty acids, such as *Bacteroides*, *Bifidobacterium*, *Clostridium* and *Roseburia* [42, 47, 48]. Contrarily, participants with the lowest plasma concentrations of OEA/PEA (Q1), showed higher abundances of *Blautia* and *Coprococcus* taxa, *C. ramosum* and *Alistipes* previously associated with obesity, inflammation and animal-based diet rich in fat and proteins [49–51]. Therefore, Q4 participants, harbouring a healthy gut microbiota and displaying lower plasma AEA and higher plasma 2-AG concentrations, might possess a more functional intestinal barrier at baseline than Q1 participants. This was further supported by the differences found in functional and metabolic features of microbial communities between Q1 and Q4. The more abundant glycocholate degradation module (MF0044) in Q4 might lower the intestinal concentration of glycocholic acid as occurring in healthy individuals vs those with IBD [52]. Similarly, the glucose (MF0057) and lactose (MF0048) degradation modules might indicate a better ability to metabolise those nutrients whose transport modules, are increased in ileal Chron’s disease [53]. Interestingly, the participants in Q4 had also a lower intake of refined-grain products, oils, fats, snacks and meat than Q1 and, switching to an MD, ameliorated the inflammatory status. Although Q4 participants in the MD group consumed more whole-grain products and less meat than those in the CT group, their snacks intake decreased only after 4 weeks. This may explain why participants in Q4 experienced the greatest ant-inflammatory benefit from the MD after 4 weeks of intervention.

The finding that Q1 participants at baseline consumed more refined-grain products, oils, fats, snacks and meat as compared to Q4 may suggest that the consumption of those foods has a role in shaping the functional and metabolic features of the gut microbiome in humans. Owing to the nature and duration of the study, these results should be interpreted cautiously as a preliminary indication of the effect of high sugar, high fat and high-energy-dense food consumption on the gut microbiome.

The macronutrient composition of the diets was not different between quartiles at baseline, suggesting that other food components in snacks, meat (including processed meat), refined-grain products, oils and fat might affect the endocannabinoid system and the gut microbiome. Evidence shows that food emulsifiers affect gut microbiota composition promoting colitis and metabolic syndrome in mice [54] while noncaloric artificial sweetener intake can cause intestinal dysbiosis in humans [55].

This study has three main strengths. First, the nutritional intervention applied allowed us to demonstrate an involvement of the endocannabinoid system in the interplay between MD consumption, the gut microbiome and health independently of changes in body weight and composition. Secondly, the influence of the baseline endocannabinoid tone in the physiological response to MD consumption provides new clinical indications for the nutritional management of subjects at risk of metabolic diseases within the framework of personalised nutrition. Thirdly, the *intention-to-treat* analysis of the gut microbiome data evidenced the effect of the MD in increasing the abundance of *A. Muciniphila*. This finding suggests that in the present study the overall aspects of MD, other than the increased intake in fruit and vegetables considered to select subjects in the *per-protocol* analysis in Meslier and co-workers [9], might contribute with the higher sample size in achieving the statistical differences between MD and CT as regards the diet-induced modulation of *A. muciniphila*.

On the other hand, since we did not measure gut permeability, we could not establish a causal role of gut barrier function/homeostasis in the interplay among the endocannabinoid system, the MD and health outcomes.

In conclusion, our study demonstrates that a switch from a Western-like diet to an isocaloric MD affects the endocannabinoid system and increases *A. muciniphila* abundance in the gut, independent of body weight changes in subjects with lifestyle risk factors for metabolic disease. Moreover, individual endocannabinoid tone at baseline drives a personalised response to the MD ameliorating insulin sensitivity or systemic inflammation. Specifically, individuals with a low-plasma OEA/PEA ratio (high AEA) exhibit an increase of plasma OEA/PEA and 2-AG, ameliorating insulin sensitivity, while those with a high-plasma OEA/PEA ratio (low AEA) show an increase in intestinal *A. muciniphila* and a reduction in systemic inflammation.

A high-plasma OEA/PEA ratio and low AEA concentration reflect a more functional gut microbiome characterised by species associated with gut homeostasis and barrier integrity. Finally, we provide preliminary evidence that the individual endocannabinoid system is shaped by habitual intake of meat, refined-grain products, oils, fat and snacks affecting gut microbiome functionality and its responsiveness to dietary changes.

Altogether our findings shed light on endocannabinoid system implication in the interplay between diet, gut and health, revealing opportunities for clinical practice in the context of personalised nutrition. Specifically, the data suggest that the measure of plasma OEA/PEA ratio and AEA concentration might mirror individual gut microbiome and intestinal barrier functionality determining the individual responsiveness towards metabolic and/or anti-inflammatory changes following a dietary intervention with an MD.

## Supplementary Information

Below is the link to the electronic supplementary material.

Supplementary file 1 (DOCX 727 KB)

## Data Availability

Data are available in a public, open access repository. Metagenomic reads generated in this study are available (without conditions of reuse) under the accession number PRJEB33500 at the European Nucleotide Archive (ENA) in EBI (accession code: PRJEB33500; https://www.ebi.ac.uk/ena/data/view/PRJEB33500). Other datasets supporting the current study are available from the corresponding author on request.

## Abbreviations

2-AG: 2-Arachidonylglycerol
AEA: Arachidonoylethanolamide or anandamide
AEAd8: Anandamide-d8
BMI: Body mass index
CRP: C-reactive protein
CT: Control group
ECs: Endocannabinoids
HDL: High-density lipoprotein
HOMA-index: Homeostatic model assessment of insulin resistance-index
IBD: Inflammatory bowel disease
IMI: Italian Mediterranean Index
LDL: Low-density lipoprotein
LEA: Linoleoylethanolamide
MD: Mediterranean diet
MPS: Metagenomic species pangenome
MRM: Multiple reaction monitoring
NAEs: N-acylethanolamines
OEA: Oleoylethanolamide
PEA: Palmitoylethanolamide
WD: Western diet

## References

[1] Franquesa M, Pujol-Busquets G, García-Fernández E, Rico L, Shamirian-Pulido L, Aguilar-Martínez A, Medina F, Serra-Majem L, Bach-Faig A (2019). Mediterranean diet and cardiodiabesity: a systematic review through evidence-based answers to key clinical questions. Nutrients.

[2] Soltani S, Jayedi A, Shab-Bidar S, Becerra-Tomás N, Salas-Salvadó J (2019). Adherence to the Mediterranean Diet in relation to all-cause mortality: a systematic review and dose-response meta-analysis of prospective cohort studies. Adv Nutr.

[3] van Ommen B, van den Broek T, de Hoogh I, van Erk M, van Someren E, Rouhani-Rankouhi T, Anthony JC, Hogenelst K, Pasman W, Boorsma A (2017). Systems biology of personalized nutrition. Nutr Rev.

[4] Zeevi D, Korem T, Zmora N, Israeli D, Rothschild D, Weinberger A, Ben-Yacov O, Lador D, Avnit-Sagi T, Suez J (2015). Personalized nutrition by prediction of glycemic responses. Cell.

[5] De Filippis F, Vitaglione P, Cuomo R, Berni Canani R, Ercolini D (2018). Dietary interventions to modulate the gut microbiome—how far away are we from precision medicine. Inflamm Bowel Dis.

[6] Kolodziejczyk, A. A., Zheng, D., Elinav, E. (2019). Diet–microbiota interactions and personalized nutrition. Nat Rev Microbiol, 1–12 10.1038/s41579-019-0256-8 31541197

[7] Laiola M, De Filippis F, Vitaglione P, Ercolini D (2020). A Mediterranean diet intervention reduces the levels of salivary periodontopathogenic bacteria in overweight and obese subjects. Appl Environ Microbiol.

[8] Vitale M, Giacco R, Laiola M, Della Pepa G, Luongo D, Mangione A, Salamone D, Vitaglione P, Ercolini D, Rivellese AA (2020). Acute and chronic improvement in postprandial glucose metabolism by a diet resembling the traditional Mediterranean dietary pattern: Can SCFAs play a role?. Clin Nutr..

[9] Meslier V, Laiola M, Roager HM, De Filippis F, Roume H, Quinquis B, Pasolli E, Rivellese A, Dragsted LO, Vitaglione P (2020). Mediterranean diet intervention in overweight and obese subjects lowers plasma cholesterol and causes changes in the gut microbiome and metabolome independently of energy intake. Gut.

[10] Ghosh TS, Rampelli S, Jeffery IB, Santoro A, Neto M, Capri M, Giampieri E, Jennings A, Candela M, Turroni S, Zoetendal EG (2020). Mediterranean diet intervention alters the gut microbiome in older people reducing frailty and improving health status: the NU-AGE 1-year dietary intervention across five European countries. Gut.

[11] Tilg H, Zmora N, Adolph TE, Elinav E (2020). The intestinal microbiota fuelling metabolic inflammation. Nat Rev Immunol.

[12] Everard A, Belzer C, Geurts L, Ouwerkerk JP, Druart C, Bindels LB, Guiot Y, Derrien M, Muccioli GG, De Vos WM (2013). Cross-talk between Akkermansia muciniphila and intestinal epithelium controls diet-induced obesity. Proc Natl Acad Sci.

[13] Plovier H, Everard A, Druart C, Depommier C, Van Hul M, Geurts L, Chilloux J, Ottman N, Duparc T, Lichtenstein L (2017). A purified membrane protein from Akkermansia muciniphila or the pasteurized bacterium improves metabolism in obese and diabetic mice. Nat Med.

[14] Everard A, Plovier H, Rastelli M, Van Hul M, de Wouters d’Oplinter A, Geurts L, Druart C, Robine S, Delzenne N, Muccioli GG (2019). Intestinal epithelial N-acylphosphatidylethanolamine phospholipase D links dietary fat to metabolic adaptations in obesity and steatosis. Nat Commun.

[15] Cani PD, Plovier H, Van Hul M, Geurts L, Delzenne NM, Druart C, Everard A (2016). Endocannabinoids—at the crossroads between the gut microbiota and host metabolism. Nat Rev Endocrinol.

[16] Hillard CJ (2018). Circulating endocannabinoids: from whence do they come and where are they going?. Neuropsychopharmacology.

[17] Witkamp R (2018). The role of fatty acids and their endocannabinoid-like derivatives in the molecular regulation of appetite. Mol Aspects Med.

[18] Silvestri C, Di Marzo V (2013). The endocannabinoid system in energy homeostasis and the etiopathology of metabolic disorders. Cell Metab.

[19] Fanelli F, Mezzullo M, Repaci A, Belluomo I, Gasparini DI, Di Dalmazi G, Mastroroberto M, Vicennati V, Gambinieri A, Pasquali R (2018). Profiling plasma N-Acylethanolamine levels and their ratios as a biomarker of obesity and dysmetabolism. Mol Metab.

[20] Mennella I, Ferracane R, Zucco F, Fogliano V, Vitaglione P (2015). Food liking enhances the plasma response of 2-arachidonoylglycerol and of pancreatic polypeptide upon modified sham feeding in humans. J Nutr.

[21] Mennella I, Savarese M, Ferracane R, Sacchi R, Vitaglione P (2015). Oleic acid content of a meal promotes oleoylethanolamide response and reduces subsequent energy intake in humans. Food Funct.

[22] Tagliamonte S, Gill CI, Pourshahidi LK, Slevin MM, Price RK, Ferracane R, Lawther R, O’Connor G, Vitaglione P (2020). Endocannabinoids, endocannabinoid-like molecules and their precursors in human small intestinal lumen and plasma: does diet affect them?. Eur J Nutr.

[23] Trichopoulou A, Martínez-González MA, Tong TY, Forouhi NG, Khandelwal S, Prabhakaran D, Mozaffarian D, de Lorgeril M (2014). Definitions and potential health benefits of the Mediterranean diet: views from experts around the world. BMC Med.

[24] Matthews DR, Hosker JP, Rudenski AS, Naylor BA, Treacher DF, Turner RC (1985). Homeostasis model assessment: insulin resistance and β-cell function from fasting plasma glucose and insulin concentrations in man. Diabetologia.

[25] Marczylo TH, Lam PM, Nallendran V, Taylor AH, Konje JC (2009). A solid-phase method for the extraction and measurement of anandamide from multiple human biomatrices. Anal Biochem.

[26] Plaza Oñate F, Le Chatelier E, Almeida M, Cervino AC, Gauthier F, Magoulès F, Ehrlich DS, Pichaud M (2019). MSPminer: abundance-based reconstitution of microbial pan-genomes from shotgun metagenomic data. Bioinformatics.

[27] Dao MC, Everard A, Aron-Wisnewsky J, Sokolovska N, Prifti E, Verger EO, Kayser BD, Levenez F, Chilloux J, Dumas ME (2016). Akkermansia muciniphila and improved metabolic health during a dietary intervention in obesity: relationship with gut microbiome richness and ecology. Gut.

[28] Fanelli F, Mezzullo M, Belluomo I, Di Lallo VD, Baccini M, Gasparini DI, Casadio E, Mastroroberto M, Vicennati V, Gambineri A, Morselli-Labate AM (2017). Plasma 2-arachidonoylglycerol is a biomarker of age and menopause related insulin resistance and dyslipidemia in lean but not in obese men and women. Mol Metab.

[29] Le Chatelier E, Nielsen T, Qin J, Prifti E, Hildebrand F, Falony G, Arumugam M, Batto J, Almeida M, Leonard P (2013). Richness of human gut microbiome correlates with metabolic markers. Nature.

[30] Segata N, Izard J, Waldron L, Gevers D, Miropolsky L, Garrett WS, Huttenhower C (2011). Metagenomic biomarker discovery and explanation. Genome Biol.

[31] Engeli S, Lehmann AC, Kaminski J, Haas V, Janke J, Zoerner AA, Luft FC, Tsikas D, Jordan J (2014). Influence of dietary fat intake on the endocannabinoid system in lean and obese subjects. Obesity.

[32] Jones PJ, Lin L, Gillingham LG, Yang H, Omar JM (2014). Modulation of plasma N-acylethanolamine levels and physiological parameters by dietary fatty acid composition in humans. J Lipid Res.

[33] Little TJ, Cvijanovic N, DiPatrizio NV, Argueta DA, Rayner CK, Feinle-Bisset C, Young RL (2018). Endocannabinoids and cannabinoid receptors as regulators of endocrine functions and tissue metabolism: plasma endocannabinoid levels in lean, overweight, and obese humans: relationships to intestinal permeability markers, inflammation, and incretin secretion. Am J Physiol Endocrinol Metab.

[34] Grill M, Högenauer C, Blesl A, Haybaeck J, Golob-Schwarzl N, Ferreirós N, Thomas D, Gurke R, Trötzmüller M, Gallé B, g, (2019). Members of the endocannabinoid system are distinctly regulated in inflammatory bowel disease and colorectal cancer. Sci Rep.

[35] Cani PD, Amar J, Iglesias MA, Poggi M, Knauf C, Bastelica D, Neyrinck AM, Fava F, Tuohy KM, Waget A (2007). Metabolic endotoxemia initiates obesity and insulin resistance. Diabetes.

[36] Cani PD, de Vos WM (2017). Next-generation beneficial microbes: the case of Akkermansia muciniphila. Front Microbiol.

[37] Yassour M, Lim MY, Yun HS, Tickle TL, Sung J, Song YM, Lee K, Franzosa AE, Morgan XC, Lander E (2016). Sub-clinical detection of gut microbial biomarkers of obesity and type 2 diabetes. Genome Med.

[38] Li J, Zhao F, Wang Y, Chen J, Tao J, Tian G, Wu S, Liu W, Cui Q, Zhang W (2017). Gut microbiota dysbiosis contributes to the development of hypertension. Microbiome.

[39] Forslund K, Hildebrand F, Nielsen T, Falony G, Le Chatelier E, Sunagawa S, Prifti E, Vieira-Silva S, Gudmundsdottir V, Arumugam M (2015). Disentangling type 2 diabetes and metformin treatment signatures in the human gut microbiota. Nature.

[40] Greer RL, Dong X, Moraes ACF, Zielke RA, Fernandes GR, Peremyslova E, Vasquez-Perez S, Schoenborn AA, Gomes WP, Ferreira SR (2016). Akkermansia muciniphila mediates negative effects of IFNγ on glucose metabolism. Nat Commun.

[41] Machiels K, Joossens M, Sabino J, De Preter V, Arijs I, Eeckhaut V, Ballet V, Claes K, Immerseel FV, Ferrante M (2014). A decrease of the butyrate-producing species *Roseburia hominis* and *Faecalibacterium prausnitzii* defines dysbiosis in patients with ulcerative colitis. Gut.

[42] Wong CB, Odamaki T, Xiao JZ (2019). Beneficial effects of Bifidobacterium longum subsp. longum BB536 on human health: Modulation of gut microbiome as the principal action. J Funct Foods.

[43] Jiang W, Wu N, Wang X, Chi Y, Zhang Y, Qiu X, Hu Y, Liu Y (2015). Dysbiosis gut microbiota associated with inflammation and impaired mucosal immune function in intestine of humans with non-alcoholic fatty liver disease. Sci Rep.

[44] De Filippis F, Pasolli E, Tett A, Tarallo S, Naccarati A, De Angelis M, Neviani E, Cocolin L, Gobbetti M, Segata N (2019). Distinct genetic and functional traits of human intestinal Prevotella copri strains are associated with different habitual diets. Cell Host Microbe.

[45] Saltzman ET, Palacios T, Thomsen M, Vitetta L (2018). Intestinal microbiome shifts, dysbiosis, inflammation, and non-alcoholic fatty liver disease. Front Microbiol.

[46] De Filippis F, Pellegrini N, Laghi L, Gobbetti M, Ercolini D (2016). Unusual sub-genus associations of faecal Prevotella and Bacteroides with specific dietary patterns. Microbiome.

[47] Tamanai-Shacoori Z, Smida I, Bousarghin L, Loreal O, Meuric V, Fong SB, Bonnaure-Mallet M, Jolivet-Gougeon A (2017). *Roseburia* spp.: a marker of health?. Future Microbiol.

[48] Alou MT, Lagier JC, Raoult D (2016). Diet influence on the gut microbiota and dysbiosis related to nutritional disorders. Human Microbiome J.

[49] Castaner O, Goday A, Park YM, Lee SH, Magkos F, Shiow SATE, Schröder H (2018). The gut microbiome profile in obesity: a systematic review. Int J Endocrinol..

[50] Woting A, Pfeiffer N, Loh G, Klaus S, Blaut M (2014). Clostridium ramosum promotes high-fat diet-induced obesity in gnotobiotic mouse models. MBio.

[51] Roager HM, Vogt JK, Kristensen M, Hansen LBS, Ibrügger S, Mærkedahl RB, Bahl MI, Lind MV, Nielsen RL, Gøbel RJ (2019). Whole grain-rich diet reduces body weight and systemic low-grade inflammation without inducing major changes of the gut microbiome: a randomised cross-over trial. Gut.

[52] Das P, Marcišauskas S, Ji B, Nielsen J (2019). Metagenomic analysis of bile salt biotransformation in the human gut microbiome. BMC Genomics.

[53] Morgan XC, Tickle TL, Sokol H, Gevers D, Devaney KL, Ward DV, Reyes JA, Shah SA, LeLeiko N, Bousvaros A (2012). Dysfunction of the intestinal microbiome in inflammatory bowel disease and treatment. Genome Biol.

[54] Chassaing B, Koren O, Goodrich JK, Poole AC, Srinivasan S, Ley RE, Gewirtz AT (2015). Dietary emulsifiers impact the mouse gut microbiota promoting colitis and metabolic syndrome. Nature.

[55] Suez J, Korem T, Zeevi D, Zilberman-Schapira G, Thaiss CA, Maza O, Israeli D, Zmora N, Gilad S, Kuperman Y (2014). Artificial sweeteners induce glucose intolerance by altering the gut microbiota. Nature.

