# Supplementary material for: Mediterranean diet consumption affects the endocannabinoid system in overweight and obese subjects: possible links with gut microbiome, insulin resistance and inflammation
Source: Eur J Nutr. 2021 Mar 24;60(7):3703–16. doi: 10.1007/s00394-021-02538-8 (PMC8437855; doi:10.1007/s00394-021-02538-8)
Supplement: Supplementary file 1 — (DOCX 727 KB) [file 394_2021_2538_MOESM1_ESM.docx]

**Electronic Supplementary Material**

**Mediterranean diet consumption affects the endocannabinoid system in overweight and obese subjects: possible links with gut microbiome, insulin resistance and inflammation**

*European Journal of Nutrition*

Silvia Tagliamonte, Manolo Laiola, Rosalia Ferracane, Marilena Vitale, Maria A. Gallo, Victoria Meslier, Nicolas Pons, Danilo Ercolini, Paola Vitaglione*

***Corresponding author:**

Paola Vitaglione

Department of Agricultural Sciences, University of Naples “Federico II”

Parco Gussone Ed. 84, Portici (NA), 80055, Italy

Telephone: +39 081 2539357;

E-mail: [*paola.vitaglione@unina.it*](mailto:paola.vitaglione@unina.it)

**Supplementary Table 1.** Acquisition parameters used for the LC/MS/MS analysis.

|  | Precursor ion [M+H]^+^  *m/z* | Precursor ion [M+H]^+^ *m/z* | DP | CE |
| --- | --- | --- | --- | --- |
| AEA ^a^ | 348.0 | 62 | 35 | 35 |
| OEA ^b^ | 326.0 | 62 | 60 | 35 |
| LEA ^c^ | 324.0 | 62 | 60 | 30 |
| PEA ^d^ | 300.0 | 62 | 60 | 30 |
| 2-AG ^e^ | 379.4 | 287.3 | 38 | 15 |
|  |  | 203.5 |  | 25 |
| AEA-d8 ^f^ | 356.5 | 63.2 | 50 | 31 |
|  |  | 209.3 |  | 18 |

^a^ AEA, Arachidonoylethanolamide;

^b^ OEA, Oleoylethanolamide;

^c^ LEA, Linoylethanolamide;

^d^ PEA, Palmitoylethanolamide;

^e^ 2-AG, 2-Arachidonoylglicerol;

^f^ AEA-d8, Arachidonoylethanolamide-d8.

**Supplementary Table 2.** ECs and NAEs limit of detection (LoD) and limit of quantitation (LoQ).

| Compound | LoD (ng/mL) | LoQ (ng/mL) |
| --- | --- | --- |
| Endocannabinoids |  |  |
| AEA ^a^ | 0.02 | 0.05 |
| 2-AG ^b^ | 0.25 | 0.5 |
| N-acylethanolamines |  |  |
| OEA ^c^ | 0.05 | 0.1 |
| LEA ^d^ | 0.05 | 0.1 |
| PEA ^e^ | 0.05 | 0.1 |

^a^ AEA, Arachidonoylethanolamide;

^b^ 2-AG, 2-Arachidonoylglicerol;

^c^ OEA, Oleoylethanolamide;

^d^ LEA, Linoylethanolamide;

^e^ PEA, Palmitoylethanolamide.

**Supplementary Table 3**. List of contrasted Metagenomic Species Pangenome (MSP) species between CT and MD groups after 4 weeks of intervention.

| **MSP^a^** | **Phylum** | **Species annotation** | **Enriched in** | **p-value^b^** | **q-value^c^** |
| --- | --- | --- | --- | --- | --- |
|  |  |  |  |  |  |
| msp_0121 | Firmicutes | *unclassified Oscillibacter* | CT | 0.01161 | 0.7 |
| msp_0031 | Bacteroidetes | *Bacteroides stercoris* | CT | 0.01593 | 0.7 |
| msp_0152 | Firmicutes | *Ruminococcus faecis* | CT | 0.01608 | 0.7 |
| msp_0621 | Firmicutes | *Intestinibacter bartlettii* | CT | 0.03271 | 0.7 |
| msp_0296 | Firmicutes | *unclassified Lachnospiraceae* | MD | 0.00011 | 0.1 |
| msp_0906 | Firmicutes | *Clostridium sp*. 2789STDY5834924 | MD | 0.00345 | 0.7 |
| msp_0075 | Firmicutes | *Clostridium sp.* CAG^d^:91 & *sp*. 2789STDY5834873 | MD | 0.00759 | 0.7 |
| msp_0314 | Firmicutes | *unclassified Clostridiales* | MD | 0.01139 | 0.7 |
| msp_0772 | Firmicutes | *Clostridiales bacterium* 1_7_47FAA & VE202-28 | MD | 0.01542 | 0.7 |
| msp_1143 | Firmicutes | *unclassified Oscillibacter* | MD | 0.01876 | 0.7 |
| msp_1533 | Firmicutes | *unclassified Clostridiales* | MD | 0.02046 | 0.7 |
| msp_0015 | Firmicutes | *Roseburia faecis* | MD | 0.02585 | 0.7 |
| msp_0986 | Firmicutes | *Coprococcus catus* | MD | 0.02695 | 0.7 |
| msp_0388 | Firmicutes | *Faecalibacterium prausnitzii* 3 (L2-6) | MD | 0.02741 | 0.7 |
| msp_0930 | Firmicutes | *unclassified Lachnospiraceae* | MD | 0.03152 | 0.7 |
| msp_1641 | Firmicutes | *unclassified Lachnospiraceae* | MD | 0.04004 | 0.7 |
| msp_0903 | Firmicutes | *Oscillibacter sp*. 57_20 | MD | 0.04584 | 0.7 |

^a^ after 20% occurrence filter.

^b^ P-values indicate significance for variation at 8 wk compared to baseline in MD vs CT by unpaired Wilcoxon rank sum test.

^c^ q-values after adjustment for multiple comparisons using the Benjamini-Hochberg procedure.

^d^ CAG, co-abundant genes.

**Supplementary Table 4**. List of contrasted Metagenomic Species Pangenome (MSP) species between CT and MD groups after 8 weeks of intervention.

| **MSP^a^** | **Phylum** | **Species annotation** | **Enriched in** | **p-value^b^** | **q-value^c^** |
| --- | --- | --- | --- | --- | --- |
| msp_0152 | Firmicutes | *Ruminococcus faecis* | CT | 0.00149 | 0.481 |
| msp_0510 | Firmicutes | *Clostridium glycyrrhizinilyticum* | CT | 0.00504 | 0.704 |
| msp_0058 | Firmicutes | *Ruminococcus gnavus* | CT | 0.02365 | 0.716 |
| msp_0056 | Firmicutes | *unclassified Clostridiales* | CT | 0.02565 | 0.716 |
| msp_0039 | Firmicutes | *Clostridium sp*. CAG^d^:127 | CT | 0.03142 | 0.716 |
| msp_0126 | Firmicutes | *Ruminococcus torques* | CT | 0.0317 | 0.716 |
| msp_0052 | Firmicutes | *Blautia sp.* CAG^d^:257 | CT | 0.03388 | 0.716 |
| msp_0565 | Firmicutes | *unclassified Ruminococcaceae* | CT | 0.03519 | 0.716 |
| msp_0385 | Firmicutes | *Ruminococcaceae bacterium* D16 | CT | 0.04614 | 0.716 |
| msp_0071 | Firmicutes | *Roseburia hominis* | MD | 0.00031 | 0.204 |
| msp_0296 | Firmicutes | *unclassified Lachnospiraceae* | MD | 0.00034 | 0.204 |
| msp_0906 | Firmicutes | *Clostridium sp.* 2789STDY5834924 | MD | 0.00438 | 0.704 |
| msp_0457 | Firmicutes | *Faecalibacterium sp.* CAG^d^:82 | MD | 0.00522 | 0.704 |
| msp_1533 | Firmicutes | *unclassified Clostridiales* | MD | 0.00827 | 0.716 |
| msp_0820 | Firmicutes | *unclassified Clostridiales* | MD | 0.00993 | 0.716 |
| msp_0093 | Firmicutes | *Clostridium sp.* CAG^d^:62 | MD | 0.01006 | 0.716 |
| msp_0903 | Firmicutes | *Oscillibacter sp*. 57_20 | MD | 0.01223 | 0.716 |
| msp_0360 | Firmicutes | *Clostridium asparagiforme == lavalense* | MD | 0.01697 | 0.716 |
| msp_0025 | Verrucomicrobia | *Akkermansia muciniphila* | MD | 0.026 | 0.716 |
| msp_0546 | Firmicutes | *Clostridium sp.* CAG^d^:245 | MD | 0.02425 | 0.716 |
| msp_0046 | Bacteroidetes | *Bacteroides uniformis* | MD | 0.02722 | 0.716 |
| msp_0756 | Firmicutes | *unclassified Blautia* | MD | 0.03125 | 0.716 |
| msp_1362 | Firmicutes | *Holdemania massiliensis* | MD | 0.03708 | 0.716 |
| msp_0075 | Firmicutes | *Clostridium* *sp*. CAG^d^:91 & *sp*. 2789STDY5834873 | MD | 0.0384 | 0.716 |
| msp_0473c | Firmicutes | *Clostridium sp.* 2789STDY5834874 & *sp*. 2789STDY5608885 | MD | 0.04425 | 0.716 |

^a^ after 20% occurrence filter.

^b^ P-values indicate significance for variation at 8 wk compared to baseline in MD vs CT by unpaired Wilcoxon rank sum test.

^c^ q-values after adjustment for multiple comparisons using the Benjamini-Hochberg procedure.

^d^ CAG, co-abundant genes.

**Supplementary Table 5.** Contrasting gut microbial functional modules between Q1 and Q4 quartiles at baseline.

| **GMM** | **Module definition** | **HIER1** | **Enriched in** | **p-value** | **q-value** |
| --- | --- | --- | --- | --- | --- |
| MF0005 | acetylneuraminate and acetylmannosamine degradation | amines and polyamines  degradation | Q4 | 0.001 | 0.912 |
|  |  |  |  |  |  |
| MF0048 | Lactose degradation | carbohydrate  degradation | Q4 | 0.009 | 0.912 |
|  |  |  |  |  |  |
| MF0037 | arginine degradation (AST/succinyltransferase pathway) | central metabolism | Q4 | 0.01 | 0.912 |
|  |  |  |  |  |  |
| MF0057 | alpha-D-glucose and alpha-D-glucose 1-phosphate degradation | carbohydrate  degradation | Q4 | 0.01 | 0.912 |
|  |  |  |  |  |  |
| MF0053 | allose degradation | carbohydrate  degradation | Q4 | 0.02 | 0.912 |
|  |  |  |  |  |  |
| MF0044 | glycocholate degradation | Bile acid metabolism | Q4 | 0.04 | 0.912 |

Q1= minimum to 25^th^ percentile of OEA/PEA ratio at baseline (CT, n= 9; MD, n=11), Q4= 75^th^ percentile to maximum of OEA/PEA ratio at baseline (CT, n=10; MD, n=10). P-values refer to Wilcoxon rank sum tests and q-values to adjustment for multiple comparisons using Benjamini-Hochberg procedure.

**Supplementary Table 6.** Anthropometrical variables, body composition, and nutritional composition of diets of participants in Q1 and Q4 at baseline (0 wk).

|  | **Q1 (n=20)** |  | **Q4 (n=20)** |  |  |
| --- | --- | --- | --- | --- | --- |
|  | **CT(n=9) + MD (n=11)** |  | **CT(n=10) + MD(n=10)** |  | **p-value^*^** |
| Body weight, kg | 89.3 ± 3.2 |  | 86.9 ± 3.3 |  | 0.603 |
| BMI, kg/m^2 a^ | 30.7 ± 0.9 |  | 31.0 ± 0.9 |  | 0.816 |
| Hip circumference, cm | 112.2 ± 1.7 |  | 113.6 ± 1.9 |  | 0.345 |
| Waist circumference, cm | 104.8 ± 2.6 |  | 105.2 ± 2.8 |  | 0.917 |
| FFM, % ^b^ | 63.8 ± 5.2 |  | 68.2 ± 0.9 |  | 0.925 |
| FM, % ^c^ | 26.2 ± 2.6 |  | 31.8 ± 0.9 |  | 0.242 |
| Energy intake (kcal/day) | 1945.5 ± 173.1 |  | 1746.0 ± 145.8 |  | 0.383 |
| Carbohydrates (g/day) | 246.7 ± 24.4 |  | 218.1 ± 18.0 |  | 0.351 |
| *%Energy* | 50.1 ± 1.3 |  | 49.8 ± 1.2 |  | 0.869 |
| Sugars (g/day) | 70.2 ± 8.5 |  | 76.2 ± 8.2 |  | 0.615 |
| Dietary Fiber (g/day) | 28.8 ± 3.2 |  | 25.8 ± 2.7 |  | 0.470 |
| *%Energy* | 3.1 ± 0.3 |  | 3.0 ± 0.3 |  | 0.902 |
| Proteins (g/day) | 78.0 ± 7.8 |  | 66.3 ± 4.3 |  | 0.195 |
| *%Energy* | 16.0 ± 0.7 |  | 15.8 ± 0.6 |  | 0.760 |
| Fats (g/day) | 63.9 ± 5.3 |  | 59.4 ± 6.9 |  | 0.608 |
| *%Energy* | 30.3 ± 1.2 |  | 30.2 ± 1.4 |  | 0.891 |
| SFA (g/day) ^d^ | 24.5 ± 3.2 |  | 21.6 ± 3.7 |  | 0.563 |
| MUFA (g/day) ^e^ | 31.1 ± 1.9 |  | 29.4 ± 3.0 |  | 0.639 |
| PUFA (g/day) ^f^ | 8.3 ± 0.7 |  | 8.3 ± 0.8 |  | 0.992 |
| Alcohol (g/day) | 5.3 ± 2.3 |  | 1.8 ± 1.0 |  | 0.311 |
| *%Energy* | 1.5 *±* 0.6 |  | 0.6 ± 0.4 |  | 0.326 |
| Vegetal proteins (VP) (g/day) | 27.5 ± 3.1 |  | 24.3 ± 2.3 |  | 0.442 |
| Animal proteins (AP) (g/day) | 43.8 ± 5.3 |  | 35.1 ± 2.3 |  | 0.341 |
| VP/AP | 0.8 ± 0.1 |  | 0.8 ± 0.1 |  | 0.977 |

Data are expressed as mean ± SEM. Q1= minimum to 25^th^ percentile of OEA/PEA ratio at baseline (CT, n= 9; MD, n=11), Q4= 75^th^ percentile to maximum of OEA/PEA ratio at baseline (CT, n=10; MD, n=10).

P-values were assessed by independent-samples T Test.

^a^ BMI, body mass index;

^b^ FFM, fat-free mass;

^c^ FM, Fat mass.

^d^ SFA, saturated fatty acids;

^e^ MUFA, monounsaturated fatty acids;

^f^ PUFA, polyunsaturated fatty acids.

**Supplementary Table 7.** Daily food intake (g) within each category in participants in control (CT) and Mediterranean diet (MD) groups within Q1 and Q4, at baseline (0 wk), 4 weeks (4 wk) and 8 weeks (8 wk).

|  | **Q1** | | | | | | | | | **Q4** | | | | | | | | |  |
| --- | --- | --- | --- | --- | --- | --- | --- | --- | --- | --- | --- | --- | --- | --- | --- | --- | --- | --- | --- |
|  | **CT (n=9)** | | | **MD (n=11)** | | | **p-value^*^** | | | **CT (n=10)** | | | **MD (n=10)** | | | **p-value^*^** | | |  |
| **g/day** | **0 wk** | **4 wk** | **8 wk** | **0 wk** | **4 wk** | **8 wk** | **Δ4-0** | **Δ8-0** | **Δ8-4** | **0 wk** | **4 wk** | **8 wk** | **0 wk** | **4 wk** | **8 wk** | **Δ4-0** | **Δ8-0** | **Δ8-4** | |
| **Fruits^d^** | 146.9 ± 43.3 ^b^ | 287.3 ± 104.2 ^a^ | 241.7 ± 93.6 ^b^ | 112.2 ± 34.2^b^ | 322.2 ± 79.4^a^ | 228.3 ± 48.6^a^ | 0.460 | 0.767 | 0.673 | 113.2 ± 30.0^b^ | 239.8 ± 70.8^a^ | 157.8 ± 57.0^b^ | 206.3 ± 19.3^b^ | 265.0 ± 20.9^b^ | 293.4 ± 22.7^a^ | 0.305 | 0.398 | **0.020*** | |
| **Vegetables** | 211.0 ± 40.1 | 198.4 ± 29.8 | 184.2 ± 36.6 | 174.8 ± 23.8^b^ | 296.8 ± 35.4^a^ | 248.2 ± 45.8^ab^ | **0.031*** | 0.137 | 0.576 | 175.2 ± 44.3 | 201.7 ± 24.9 | 204.8 ± 32.8 | 273.8 ± 32.6 | 301.5 ± 49.0 | 342.9 ± 42.6 | 0.985 | 0.487 | 0.371 | |
| **WG^e^** | 5.4 ± 3.8 | 3.2 ± 3.2 | 7.9 ± 6.3 | 24.3 ± 22.6 ^b^ | 154.0 ± 51.5^a^ | 151.1 ± 47.1^a^ | **<0.001*** | **<0.001*** | 0.641 | 4.1 ± 2.4 | 9.6 ± 7.2 | 6.6 ± 6.6 | 6.0 ± 2.0 ^b^ | 88.9 ± 31.9 ^a^ | 95.4 ± 36.8 ^a^ | **0.001*** | **0.001*** | 0.537 | |
| **RG^f^** | 213.8 ± 53.8^⁑^ | 207.0 ± 49.5 | 186.3 ± 41.6 | 222.2 ± 62.1 ^a⁑^ | 64.8 ± 18.0 ^b^ | 29.9 ± 7.8 ^c^ | 0.080 | 0.295 | 0.882 | 90.2 ± 28.2 | 104.3 ± 32.7 | 108.3 ± 36.0 | 53.4 ± 16.3 ^a^ | 8.5 ± 7.1 ^b^ | 6.3 ± 6.3 ^b^ | **0.001*** | 0.315 | 0.143 | |
| **Legumes** | 12.5 ± 3.3 | 26.1 ± 7.2 | 22.1 ± 6.1 | 14.9 ± 4.5 ^b^ | 54.8 ± 14.8 ^a^ | 57.9 ± 18.0 ^a^ | **0.046*** | 0.073 | 0.910 | 13.0 ± 5.9 | 27.9 ± 10.0 | 17.8 ± 12.6 | 12.1 ± 6.4 ^b^ | 23.7 ± 9.0 ^a^ | 28.9 ± 12.0 ^a^ | 0.196 | 0.070 | 0.115 | |
| **Eggs** | 15.8 ± 5.3 | 11.6 ± 2.8 | 11.4 ± 2.0 | 5.6 ± 2.6 | 5.5 ± 2.9 | 4.7 ± 1.8 | 0.931 | 0.671 | 0.870 | 10.4 ± 5.6 | 24.7 ± 19.2 | 5.3 ± 2.3 | 1.8 ± 0.86 ^a^ | 0.24 ± 0.24 ^b^ | 0.41 ± 0.3 ^ab^ | 0.939 | 0.816 | 0.136 | |
| **Dairy products** | 108.6 ± 29.1 | 127.1 ± 25.7 | 104.0 ± 25.2 | 100.9 ± 32.4 | 56.5 ± 22.9 | 75.2 ± 32.2 | **0.031*** | 0.261 | 0.295 | 25.2 ± 12.3 | 22.6 ± 7.8 | 25.9 ± 8.6 | 56.5 ± 34.2 | 57.4 ± 29.3 | 70.1 ± 35.6 | 0.684 | 0.579 | 0.481 | |
| **Fish Products** | 38.1 ± 13.8 | 36.2 ± 13.1 | 48.3 ± 14.9 | 23.1 ± 7.4^b^ | 48.7 ± 12.8 ^a^ | 45.8 ± 13.8 ^a^ | 0.119 | 0.362 | 0.270 | 20.2 ± 7.3 | 32.8 ± 10.4 | 33.1 ± 10.0 | 12.7 ± 4.9 | 22.3 ± 7.1 | 20.1 ± 6.0 | 0.821 | 1.000 | 0.733 | |
| **Meat** | 127.0 ± 20.8^⁑^ | 102.3 ± 13.7 | 89.2 ± 13.1 | 71.5 ± 16.1 ^a⁑^ | 16.4 ± 4.4 ^b^ | 19.7 ± 6.3 ^b^ | 0.295 | 0.766 | **0.038*** | 53.0 ± 13.6 | 58.9 ± 16.5 | 54.2 ± 14.7 | 22.7 ± 7.6 ^a^ | 1.7 ± 0.8 ^c^ | 4.7 ± 1.7 ^b^ | 0.089 | 0.089 | 0.089 | |
| **Oils & Fats** | 9.1 ± 4.4 ^⁑^ | 11.5 ± 5.2 | 8.1 ± 4.1 | 3.5 ± 1.3 ^a⁑^ | 0.8 ± 0.5 ^b^ | 1.6 ± 1.0 ^ab^ | 0.208 | 0.878 | 0.665 | 0.6 ± 0.3 | 1.6 ± 0.5 | 1.6 ± 0.5 | 0.3 ± 0.2 | 0.0 ± 0.0 | 1.9 ± 1.9 | 0.176 | 0.204 | 0.376 | |
| **Coffee** | 48.0 ± 17.0 | 46.4 ± 15.0 | 42.1 ± 23.4 | 50.8 ± 20.9 | 70.1 ± 17.2 | 30.6 ± 7.3 | 0.174 | 0.879 | 0.339 | 53.4 ± 18.3 | 64.1 ± 18.7 | 72.8 ± 20.4 | 48.0 ± 16.8 | 43.6 ± 18.5 | 56.4 ± 20.9 | 0.596 | 0.384 | 0.874 | |
| **Fruit juices** | 2.7 ± 2.7 | 1.8 ± 1.8 | 19.6 ± 16.0 | 0.0 ± 0.0 | 0.0 ± 0.0 | 0.0 ± 0.0 | 1.000 | 1.000 | 0.710 | 31.4 ± 28.0 | 14.3 ± 14.3 | 12.9 ± 12.9 | 0.0 ± 0.0 | 0.0 ± 0.0 | 0.0 ± 0.0 | 0.481 | 0.481 | 0.739 | |
| **Wine** | 17.3 ± 11.5 | 2.4 ± 2.4 | 5.4 ± 4.7 | 49.7 ± 28.6 | 30.4 ± 29.1 | 42.0 ± 29.5 | 0.882 | 1.000 | 0.766 | 9.3 ± 6.4 | 11.5 ± 5.6 | 2.7 ± 2.1 | 8.9 ± 5.7 | 13.2 ± 8.5 | 7.0 ± 3.6 | 0.280 | 0.853 | 0.796 | |
| **Snacks** | 126.6 ± 31.2^⁑^ | 139.0 ± 25.3 | 148.6 ± 38.8 | 54.4 ± 18.5 ^a⁑^ | 19.5 ± 7.6 ^b^ | 16.5 ± 6.1 ^b^ | 0.104 | 0.051 | 0.660 | 76.1 ± 38.4 | 61.7 ± 22.3 | 32.5 ± 12.7 | 17.0 ± 3.9 ^a^ | 4.6 ± 1.7 ^b^ | 15.1 ± 11.6 ^ab^ | **0.030*** | 0.300 | 0.159 | |
| **Soft drinks** | 100.0 ± 36.9 | 76.5 ± 41.9 | 85.1 ± 35.2 | 42.8 ± 2.7 | 32.1 ± 12.0 | 40.3 ± 36.0 | 0.675 | 0.817 | 0.266 | 41.8 ± 24.4 | 28.0 ± 21.3 | 10.6 ± 33.6 | 3.3 ± 2.8 | 0.0 ± 0.0 | 0.0 ± 0.0 | 0.798 | 0.425 | 0.136 | |
| **IMI ^g^** | 4.4 ± 0.5 | 3.6 ± 0.4 | 4.0 ± 0.6 | 4.8 ± 0.6 ^b^ | 7.2 ± 0.5 ^a^ | 7.3 ± 0.6 ^a^ | **0.001*** | **0.009*** | 0.653 | 4.2 ± 0.7 | 4.4 ± 0.7 | 4.0± 0.6 | 6.1 ± 0.7 ^b^ | 8.3 ± 0.4 ^a^ | 8.4 ± 0.4 ^a^ | 0.073 | **0.027*** | 0.137 | |

Data are expressed as mean ± SEM. Q1= minimum to 25^th^ percentile of OEA/PEA ratio at baseline (CT, n= 9; MD, n=11), Q4= 75^th^ percentile to maximum of OEA/PEA ratio at baseline (CT, n=10; MD, n=10). Different letters in the row indicate statistical differences between time points within group by Wilcoxon test or 2-way ANOVA repeated measures depending on data normal distribution. *p<0.05 pairwise time points (Δ) between CT and MD in the same quartile, by independent-samples T Test or Mann-Whitney test depending on data normal distribution.

⁑ p<0.05 between all participants (MD+CT) in Q1 vs Q4 at baseline by Mann-Whitney test adjusted for energy intake.

^d^ Fruits: sum of fruits and nuts;

^e^ WG, Whole-grain products;

^f^ RG, Refined-grain products;

^g^ IMI, Italian Mediterranean Index


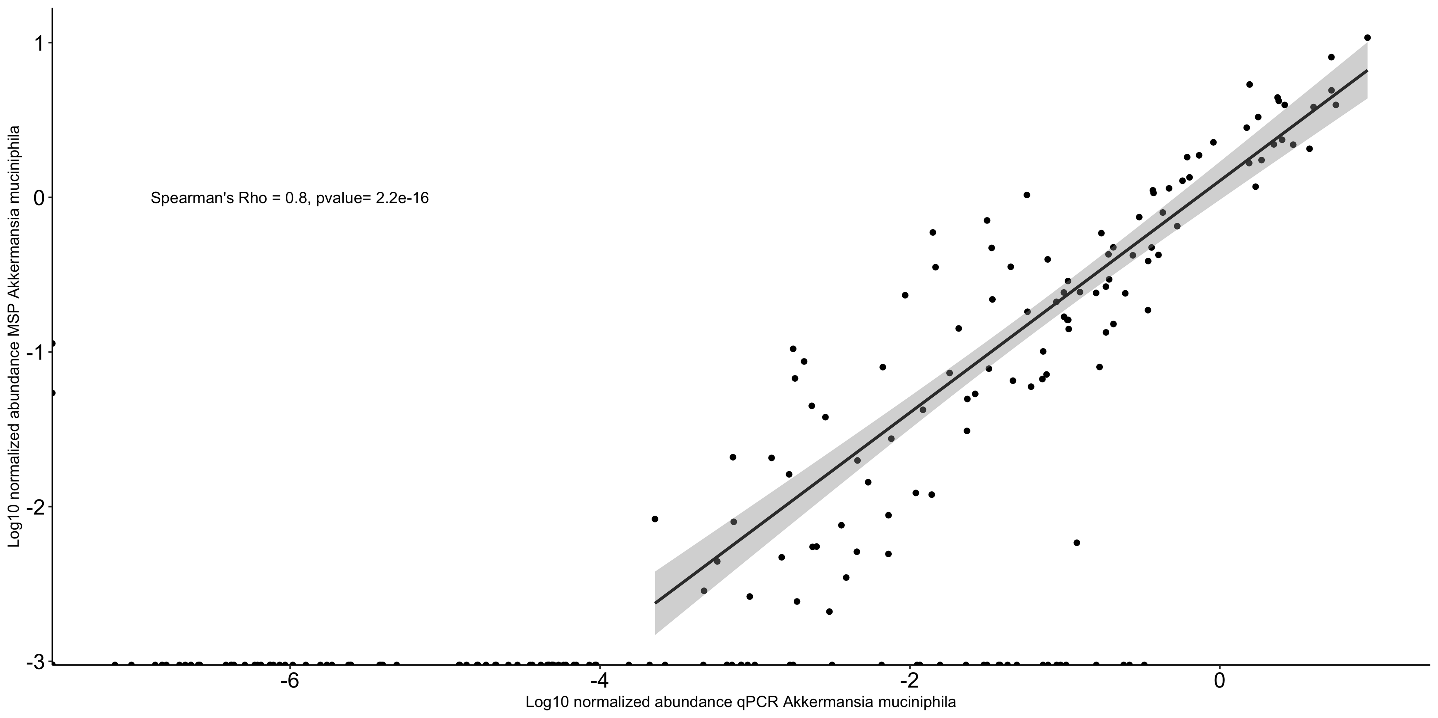


**Supplementary Fig. 1.** Spearman's rank-order correlation of Log10 normalized abundance of *Akkermansia muciniphila* between quantitative real-time PCR (qPCR) and the Metagenomic Species Pangenome (MSP). MSP, Metagenomic Species Pangenome.


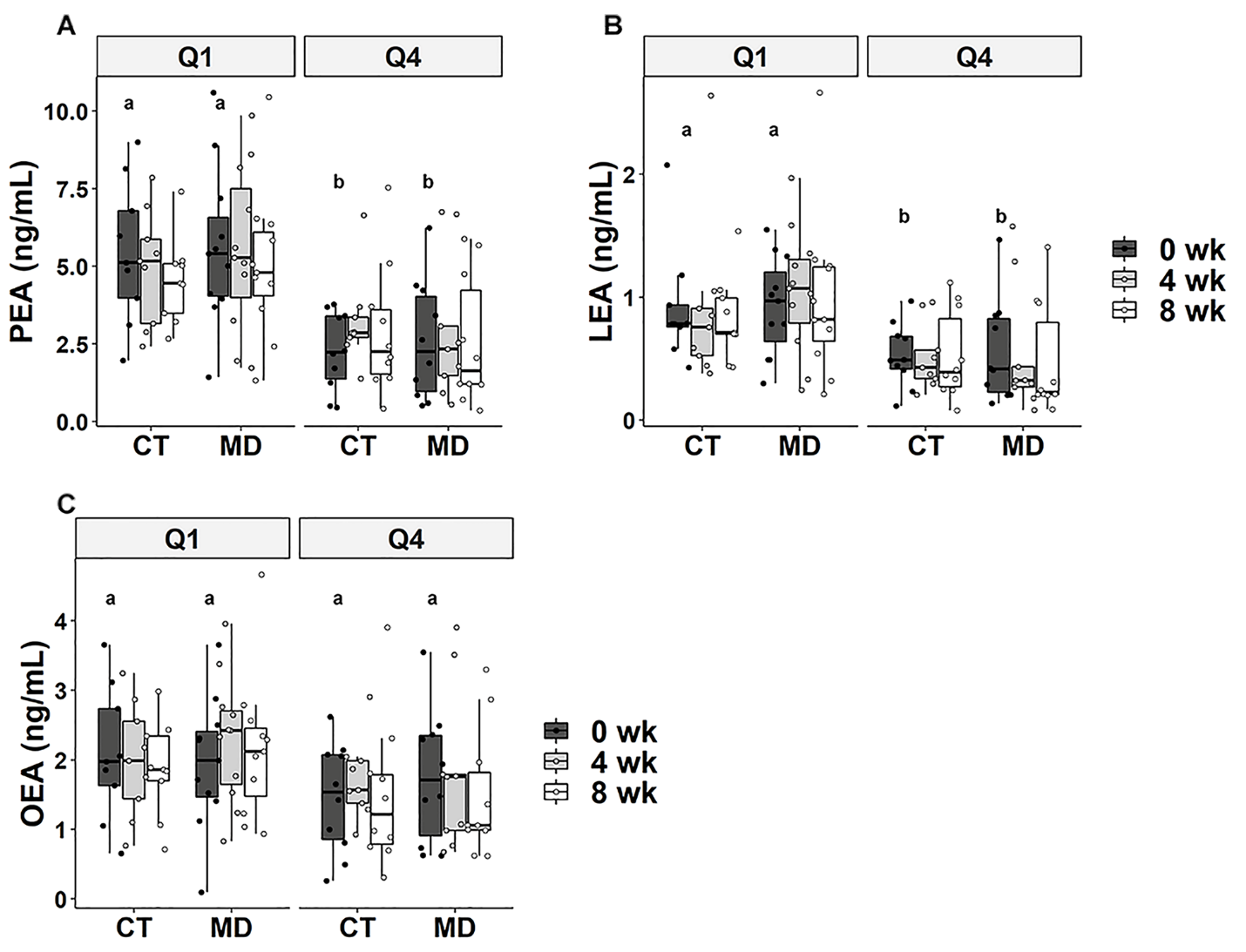


**Supplementary Fig. 2:** Plasma PEA (A) LEA (B) and OEA (C) of the control group (CT) and Mediterranean diet group (MD) in the lowest (Q1) and highest quartile (Q4) at baseline (0 wk), 4 weeks (4 wk) and 8 weeks (8 wk). Q1= minimum to 25^th^ percentile of OEA/PEA ratio at baseline (CT, n= 9; MD, n=11), Q4= 75^th^ percentile to maximum of OEA/PEA ratio at baseline (CT, n=10; MD, n=10).

Different letters on the box plots indicate p-value<0.05 between quartiles at 0 wk by independent-samples T test.

PEA, Palmitoylethanolamide; LEA, Linoylethanolamide; OEA, Oleoylethanolamide.
